# Safety and efficacy of a novel double-lumen tracheal tube in neonates with RDS: A prospective cohort study

**DOI:** 10.3389/fped.2022.1032044

**Published:** 2022-12-05

**Authors:** Chuanfeng Li, Yuxuan Du, Kaiting Yang, Huiling Cao, Hong Yang, ChunXiu Zhang, Xiongbin Li, Xingmei Deng, Yuan Shi

**Affiliations:** ^1^ Department of Neonatology, Qujing Maternity and Child Healthcare Hospital, Yunnan, China; ^2^ Department of Neonatology, Children’s Hospital of Chongqing Medical University, Chongqing, China; ^3^ National Clinical Research Center for Child Health and Disorders, Chongqing, China; ^4^ Ministry of Education Key Laboratory of Child Development and Disorders, Chongqing, China; ^5^ China International Science and Technology Cooperation Base of Child Development and Critical Disorders, Chongqing, China; ^6^ Chongqing Key Laboratory of Pediatrics, Chongqing, China

**Keywords:** newborn resuscitation, endotracheal intubation, invasive mechanical ventilation, respiratory distress syndrome, surfactant replacement therapy

## Abstract

**Background:**

The purpose of this study was to assess the safety and efficacy of a new double-lumen tracheal tube for neonates, with a conventional tracheal tube as a control.

**Method:**

Newborns with respiratory distress syndrome (RDS) requiring endotracheal intubation admitted to the tertiary neonatal intensive care unit (NICU) of Qujing Maternal and Child Healthcare Hospital in Yunnan Province between March 2021 and May 2022 were enrolled in this prospective cohort study. Outcome indicators related to effectiveness included mainly the number of intubations, duration of ventilation, duration of oxygenation, and length of stay; safety indicators included any clinical adverse effects during and after intubation. Appropriate stratified and subgroup analyses were performed according to the purpose of intubation, gestational age, and whether the drug was administered *via* endotracheal tube.

**Result:**

A total of 101 neonates were included and divided into two groups based on the choice of tracheal tube: the conventional (*n* = 50) and new (*n* = 51) tracheal tube groups. There was no statistical difference between the two groups in terms of adverse effects during and after intubation (*p* > 0.05). In neonates who were mechanically ventilated without endotracheal surfactant therapy or newborns receiving InSurE technique followed by non-invasive ventilation, no significant differences were found between the two groups regarding any of the efficacy indicators (*p* > 0.05). However, for neonates on invasive mechanical ventilation, the new tracheal tube allowed for a significant reduction in the duration of mechanical ventilation (96.50[74.00, 144.00] vs. 121.00[96.00, 196.50] hours, *p* = 0.037) and total ventilation (205.71 ± 80.24 vs. 277.56 ± 117.84 h, *p* = 0.027), when used as a route for endotracheal drug delivery. Further analysis was performed according to gestational age for newborns requiring intratracheal surfactant administration during mechanical ventilation, and the data showed that for preterm infants, the new tracheal tube not only shortened the duration of mechanical ventilation (101.75 ± 39.72 vs. 155.50 ± 51.49 h, *p* = 0.026) and total ventilation (216.00 ± 81.60 vs. 351.50 ± 113.79 h, *p* = 0.010), but also demonstrated significant advantages in reducing the duration of oxygen therapy (9.75 ± 6.02 vs. 17.33 ± 8.43 days, *p* = 0.042); however, there was no statistical difference in efficacy outcomes between the two groups in full-term infants (*p* > 0.05).

**Conclusion:**

The efficacy and safety of this new tracheal tube are promising in neonates with RDS, especially those requiring surfactant administration *via* a tracheal tube during mechanical ventilation. Given the limitations of this study, however, the clinical feasibility of this catheter needs to be further confirmed in prospective randomized trials with larger sample sizes.

**Clinical Trial Registration:**

http://www.chictr.org.cn/showproj.aspx?proj=122073

## Introduction

The significance of surfactant replacement therapy, as a cornerstone in the treatment of respiratory distress syndrome (RDS), has been well documented (1–3). Conventional administration of exogenous surfactants requires coordination with an endotracheal tube, meaning that the newborn may face temporary interruptions in ventilation while receiving therapeutic surfactants, which may lead to regurgitation of expiratory-phase surfactants and prolonged duration of ventilation.

The effects of prolonged ventilation may be particularly pronounced in neonates receiving invasive mechanical ventilation (IMV). After all, while significantly improving neonatal survival, invasive mechanical ventilation may cause a variety of adverse effects, including ventilator-related lung injury, pulmonary infections, bronchopulmonary dysplasia (BPD), and even distant neurological abnormalities (4, 5). In response to these potential risks, several emerging technologies featuring non-invasiveness or minimally-invasiveness have emerged, represented by Less Invasive Surfactant Administration (LISA), Minimally Invasive Surfactant Therapy (MIST), aerosolisation administration, and laryngeal mask administration (6–10). However, these modes of administration are not fully applicable in neonates with inadequate initial respiratory drive or failure of non-invasive respiratory support (11), and there are substantial gaps in the clinical evidence regarding these techniques except for LISA (12). Therefore, traditional administration *via* endotracheal tube during mechanical ventilation remains an irreplaceable option. Unfortunately, clinical information on modified endotracheal tubes for neonates has been rather limited to date.

Considering the limitations of the existing traditional tracheal tube used in neonates and the lack of relevant clinical information, we developed a new dual-lumen tracheal tube. By designing an additional, thinner drug delivery tube within the wall of the dominant tube, we have artificially separated the inlet and most of the route of drug and airflow delivery, avoiding temporary interruptions in mechanical ventilation during drug administration. Furthermore, the drug delivery tube does not meet the dominant tube until it extends to the superior end of the Murphy Eye. In theory, such a design can not only avoid drug residue but also reduce the risk of the Murphy Eye becoming clogged with sputum or other secretions. However, these ideas are just speculation without the support of clinical evidence. To investigate the clinical safety and efficacy of the new double-lumen tracheal tube, we conducted a prospective cohort study in newborns with RDS, using a conventional tracheal tube as a control.

## Materials and methods

### Study design

This is a single-center prospective cohort study conducted from March 2021 to May 2022 in the tertiary neonatal intensive care unit (NICU) of Qujing Maternal and Child Healthcare Hospital, Yunnan Province, China. The study complied with the principles of the Declaration of Helsinki and was approved by the Medical Ethics Committee (Approval serial number: QJFYLL2021-KY001), with a protocol registered at www.chictr.org.cn (ChiCTR2100043565).

### Participants

All newborns diagnosed with RDS and prepared for initial postnatal tracheal intubation were eligible for enrollment, including those ready for invasive mechanical ventilation or the InSurE (intubation, surfactant, extubation) technique. The choice of tracheal tube type would be determined by the operating physician. Physicians trained in the clinical operation of the new tracheal tube would choose a double-lumen tube, while those without relevant training would apply a conventional tracheal tube. The physician-patient pairing is not influenced by personal preference, but by pre-defined scheduling rules within the ward. The diagnosis of RDS would be based on clinical manifestations and chest imaging features. Typical clinical manifestations include respiratory distress, tachypnea, nasal flaring, groan and cyanosis presenting within the first 24 h of life, while chest imaging features are represented by granular shadows, air bronchograms and white lungs (13). However, patients meeting any of the following criteria would be excluded: (1) endotracheal intubation was required for reasons other than RDS, (2) received surfactant administration prior to enrollment, (3) received both mechanical ventilation and InSurE technique during hospitalization, (4) severe congenital malformations, known complex congenital heart disease or chromosomal abnormalities, (5) intraventricular hemorrhage (IVH) of grade 3 or 4, (6) transferred to another medical facility during hospitalization. Written informed consent was given by the guardians of all infants.

### Double-lumen tracheal tube

**Figure 1:**
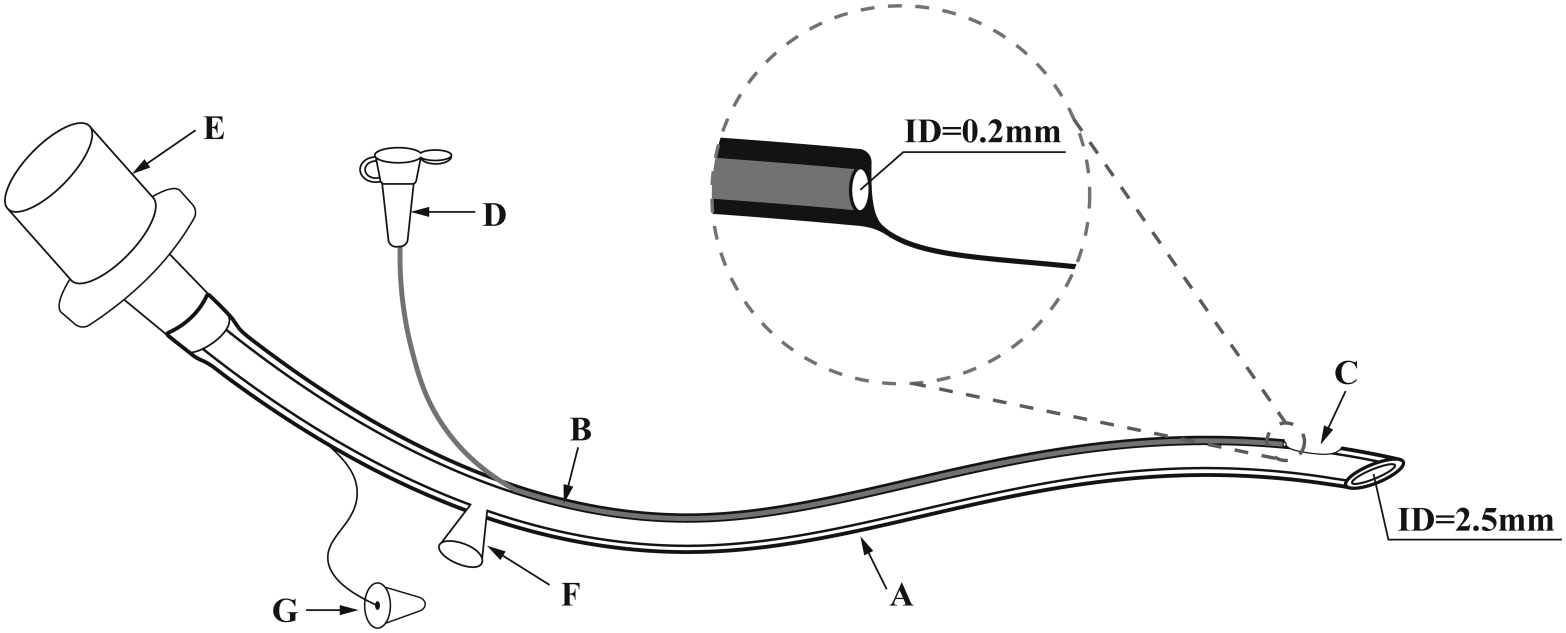
The novel doublt-lumen tracheal tube (take model 1 as an example.) ID: inner diameter. (**A**) The dominant tube, whose diameter is chosen according to the weight of the newborn. (Model 1: ID 2.5 mm; Model 2: ID 3.0 mm; Model 3: ID 3.5 mm; Model 4: ID 4.0 mm). (**B**) The drug delivery tube located inside the wall of the dominant tube, with an ID of 0.2 mm. (**C**) The Murphy Eye in direct communication with distal end of the drug delivery tube. (**D**) The plastic cap for closing the drug injection port. (**E**) The tube connector. (**F**) The aspiration cannula for the insertion of the suction tube. (**G**) The hole plug for closing the aspiration cannula when sputum aspiration is not required. In order to optimize the new double-lumen tracheal tube, the design of the aspiration cannula with its accompanying hole plug was eliminated in the actual production process.

In this study, two types of tracheal catheters were available, a conventional tube and a new non-capsular double-lumen tracheal tube (Manufacturer: Henan Tuoren Medical Device Co., Ltd.), for which a national patent (Application number: CN201821495880.4) was granted (Figure 1). To date, this double-lumen tracheal tube has not been used in any neonatal center other than our NICU. The dual-lumen tracheal tube consists of a dominant tube and a smaller diameter drug delivery tube. The main body of the drug delivery tube is located inside the wall of the dominant tube, with the distal end opening directly into the Murphy Eye in the distal wall of the main tube. Due to the small diameter of the drug delivery tube and its design within the wall of the dominant tube, the dual-lumen setup did not thicken our catheter or place additional strain on the fragile airway of the neonate. To facilitate drug administration, the proximal end of the drug delivery tube extends outside the wall of the dominant tube, and the injection port at its tip is closed with a plastic cap. There is no difference in the intubation operation between the conventional and new tracheal tubes. The diameter of the tracheal tube would be selected according to the weight of the newborn (Table 1). Additional information on the properties of the double-lumen tracheal tube can be found in Supplementary Tables S1, S2.

**Table 1:** Selection criteria for the diameter of the tracheal tube.

Weight of the newborn, g	Model	Outer diameter of the dominant tube, mm	Inner diameter of the dominant tube, mm	Outer diameter of the drug delivery tube, mm	Inner diameter of the drug delivery tube, mm
<1,000	1	3.3	2.5	0.8	0.2
1,000-2,000	2	4.0	3.0	1.0	
2,000-3,000	3	4.7	3.5	1.2	
≥3,000	4	5.3	4.0	1.3	

The outer diameter of the drug delivery tube is equal to the outer diameter of the dominant tube minus the inner diameter of the dominant tube.

### Ventilation management

In our NICU, eligible newborns would be selected for different modes of respiratory support based on individualization and weaned from ventilation when appropriate. Regardless of the mode used, the respiratory parameters would be adjusted according to the clinical presentation of the neonate to maintain oxygen saturation (SpO2) at 90%–94% (14). The specific criteria are shown below.

#### Non-invasive ventilation

Criteria for the application of non-invasive ventilation: (1) extremely premature infants with spontaneous breathing, (2) infants at high risk of RDS, (3) arterial oxygen tension (PaO₂) < 50 mmHg (1 mmHg = 0. 133 kPa) or transcutaneous oxygen saturation (TcSO2) < 90%, when the fraction of inspired oxygen (FiO2) was set above 0.3 for oxygen *via* nasal cannula, mask or hood, (4) apnea of prematurity (15).

Using the short binasal prongs as an interface, we provided two modes of non-invasive ventilation with the support of the Infant Flow® SiPAP™ (Viasys Healthcare, Yorba Linda, CA, USA): nasal continuous positive airway pressure (NCPAP) and biphasic NCPAP (BP-NCPAP). The initial pressure of the NCPAP would be set at 5 cmH_2_O and could be adjusted between 4 and 8 cmH_2_O depending on the situation, while the initial breathing parameters for BIPAP would be set at a frequency of 10–30 breaths/min, a peak inspiratory pressure (PIP) of 15–25 cmH_2_O and a positive end-expiratory pressure (PEEP) of 4–6 cmH_2_O.

When the infant was clinically stable with a PEEP < 4 cmH_2_O and FiO_2_ < 25% for 24 h, weaning from non-invasive ventilation would be considered (16). If an infant presented with PaO_2_ < 50 mmHg or TcSO_2_ < 85% on air inhalation, oxygen would be supplied by appropriate means, including High-flow nasal cannula (HFNC), oxygen environment, and nasal cannula.

#### Invasive mechanical ventilation

Infants meeting any of the following criteria would be supported with mechanical ventilation: (1) severe respiratory acidosis lasting 2 h, (2) recurrent apnoea (Frequency >3 times/h, requiring ventilation by airbag and mask) with bradycardia (Heart rate <100 beats/min), (3) hypoxemia (FiO2 > 0.5, PaO2 < 50 mmHg, or SpO 2 < 85%) for at least 2 h (17, 18). In addition, emergency tracheal intubation would be initiated when the neonate was faced with (1) severe respiratory distress, (2) neonatal pulmonary hemorrhage, (3) cardiopulmonary arrest without effective resuscitation requiring continued ventilation and rescue, or (4) other clinical emergencies (13).

In our NICU, conventional mechanical ventilation (CMV) would be the first choice, with high-frequency ventilation (HFV) as a remedial option after the failure of CMV. With the SLE 5000 ventilator as the ventilation device, mechanical ventilation and open lung strategies were used in combination. For infants receiving CMV, respiratory parameters were set as follows: respiratory rate of 30–40 breaths/min, inspiration time of 0.4 s, PEEP of 4–8 cmH_2_O, and PIP adjusted to maintain a target tidal volume (TV) of 4–6 ml/kg. For infants on HFV, mean airway pressure (MAP) ranged from 10 to 20 cmH_2_O (1 cmH20 = 0.098 kPa), oscillation amplitude from 25 to 45 cmH_2_O, and frequency from 10 to 15 Hz (1 Hz = 60 times/min), while inspiratory time was set at 33%.

The following are pre-defined criteria used to guide extraction and withdrawal: (1) weaning from CMV: RR < 30 beats/min, PIP < 16 cmH_2_O, PEEP < 5 cmH_2_O, and FiO2 < 30%, (2) weaning from HFV: MAP < 8 cm H2O, and FiO2 < 0.30 (19). In brief, extubation may be attempted when the infant on mechanical ventilation remains clinically stable with adequate spontaneous respiratory effort and FiO2 ≤ 0.30 for 6 h, followed by appropriate non-invasive ventilation or oxygen therapy support, with the specific criteria as described above (20).

### Surfactant management

When FiO_2_ was adjusted above 0.4 to maintain target SpO2, surfactant would be administered through an endotracheal tube. No more than four doses would be administered (16, 21).

The appropriate amount of surfactant (Curosurf, 200 mg/kg) with approximately 0.5 ml of air was dripped into the trachea of newborns in the supine position, *via* the catheter port (of a conventional tracheal tube) or the drug injection port (of a new tracheal tube), within 1 min under the push of a syringe. After administration, the infant's head was slightly elevated to reduce the risk of drug reflux (22). Avoid endotracheal suctioning for 1 h after administration unless there were obvious signs of endotracheal tube obstruction (22). After the endotracheal administration, the respiratory parameters would be flexibly adjusted in real-time to ensure the target oxygen saturation (23).

The InSurE technique would be used in infants receiving non-invasive ventilation, a group where pausing ventilation and tracheal intubation are mandatory steps in the medication administration, regardless of the type of tracheal tube chosen. However, temporary interruption of ventilation could be avoided for infants who were on invasive mechanical ventilation prior to drug administration, provided a new tracheal tube was chosen. In this specific population, endotracheal surfactant therapy through the injection port was administered while maintaining normal operation of the invasive ventilator, during which the neonate's clinical manifestations and oxygen saturation were monitored, and respiratory parameters could be adjusted accordingly if necessary. However, if a conventional catheter was chosen for a newborn on mechanical ventilation, a temporary interruption of ventilation was still unavoidable. After all, in this case, the ventilator must be withdrawn to make way for the drug drip until the end of the administration.

### Caffeine management

All premature newborns with a gestational age of fewer than 32 weeks and newborns presenting with apnea would be given caffeine (Caffeine Citrate Injection, Chiesi Pharmaceuticals, Parma, Italy; loading dose of 20 mg/kg with a maintenance dose of 5 mg/kg/day) (14, 24).

### The endpoint of the study

Observation of an enrolled infant would end if one of the following conditions was met: (1) death; (2) parental decision not to continue participation; (3) 1 month after discharge from the hospital.

### Data collection and definition

Prior to the study, nurses in our NICU were given uniform instructions on the clinical care of conventional and new tracheal tubes, and willing operating physicians were given uniform training on the selection protocol for the new catheter type and the clinical manipulation of endotracheal surfactant administration through the new catheter. Physicians completing the training would use the new dual-lumen tracheal tube in the study, whereas physicians without the training were only qualified to apply the conventional tracheal tube. In addition, prior to the study, all medical staff agreed on criteria for the selection of ventilation mode, the regulation of respiratory parameters, the administration of endotracheal surfactant, and the assessment of outcome indicators.

#### Baseline characteristics of neonates and the operation of the first endotracheal intubation

Baseline data on the newborns, including gestational age (GA), birth weight, sex, Apgar score, history of antenatal steroids, mode of delivery, as well as maternal risk factors during pregnancy and delivery, would be collected through the electronic medical record system. Gestational age estimates were based on obstetric information, and the rest of the demographic information was primarily obtained through guardian dictation. Furthermore, data on the operation of the first endotracheal intubation (tube diameter, depth of tube insertion through the mouth, the experience of the performing physician, and whether an endotracheal medication was performed) were collected prospectively.

#### Safety

The safety of the tracheal tube was thoroughly evaluated based on the adverse reactions of neonates during and after intubation. Adverse reactions during intubation included drug reflux, oral mucosal injury, bleeding airway injury, respiratory depression, bradycardia (Heart rate <100 beats/min for at least 10 s), tachycardia (Heart rate >160 beats/min for at least 10 s), arterial hypotension, laryngospasm, blockage of the drug delivery tube, and distortion of the dominant tube. Adverse reactions after intubation were defined as coughing and wheezing, recurrent choking on milk, pulmonary hemorrhage, pneumothorax, emphysema, intraventricular hemorrhage grade 3 or 4, secondary infection, and concurrent laryngospasm. In this study, respiratory depression was used to describe weak and irregular breathing during intubation, as evidenced by a respiratory rate of <10 breaths/min and an oxygen saturation of <80%. Arterial hypotension was defined as a mean arterial pressure in mm Hg less than the gestational age measured in weeks (25). Blockage of the drug delivery tube was represented by laborious and poor drug injection. The diagnosis of pneumothorax and emphysema was determined by chest radiograph findings, while the grading of intraventricular hemorrhage relied on intracranial ultrasound and imaging.

#### Efficacy

The following outcomes were recorded as indicators to assess the effectiveness of the tracheal tube: the total number of intubations, duration of ventilation, duration of oxygen therapy, length of hospital stay, whether discharged with oxygen, and whether presenting with recurrent dyspnea one month after discharge. For infants receiving mechanical ventilation, the duration of invasive ventilation and non-invasive ventilation after withdrawal were calculated separately, and the duration of total ventilation was defined as the sum of both. The record of oxygen supply ended at the discharge, and home oxygen therapy (HOT) was not included. All infants would be assessed for recurrent respiratory distress one month after discharge by telephone follow-up.

### Statistical analysis

Based on previous studies, the incidence of intraventricular hemorrhage (grade 3 or 4) after intubation with a conventional tracheal tube was approximately 17% (26). Assuming that the application of the new dual-lumen catheter would result in a 16% reduction in this risk, a minimum of 47 neonates per group was calculated by PASS software (version 15.0) at a 0.05 two-sided significance and an 80% power. Data analysis would be performed using SPSS statistical software (version 26.0). Normally distributed continuous variables would be presented as mean ± standard deviation (SD) (Range) and compared between groups using the independent-samples T test, while non-normally distributed continuous variables would be expressed as median [interquartile range (IQR)] and analyzed using the Mann–Whitney *U* test. For dichotomous variables represented as number (%), the *χ*^2^ test or Fisher's exact test would be chosen for the analysis. When the *P*-value was less than 0.05, statistical significance could be considered. Stratified analysis according to the purpose of intubation (Invasive mechanical ventilation and the InSurE technique) and gestational age (Full-term and preterm) would be conducted using the Cochran-Mantel-Haenszel test. When necessary, subgroup analysis of the primary outcomes would also be performed based on the reason for intubation (InSurE technique or invasive mechanical ventilation), gestational age, and whether the drug was administered endotracheally.

## Result

**Figure 2:**
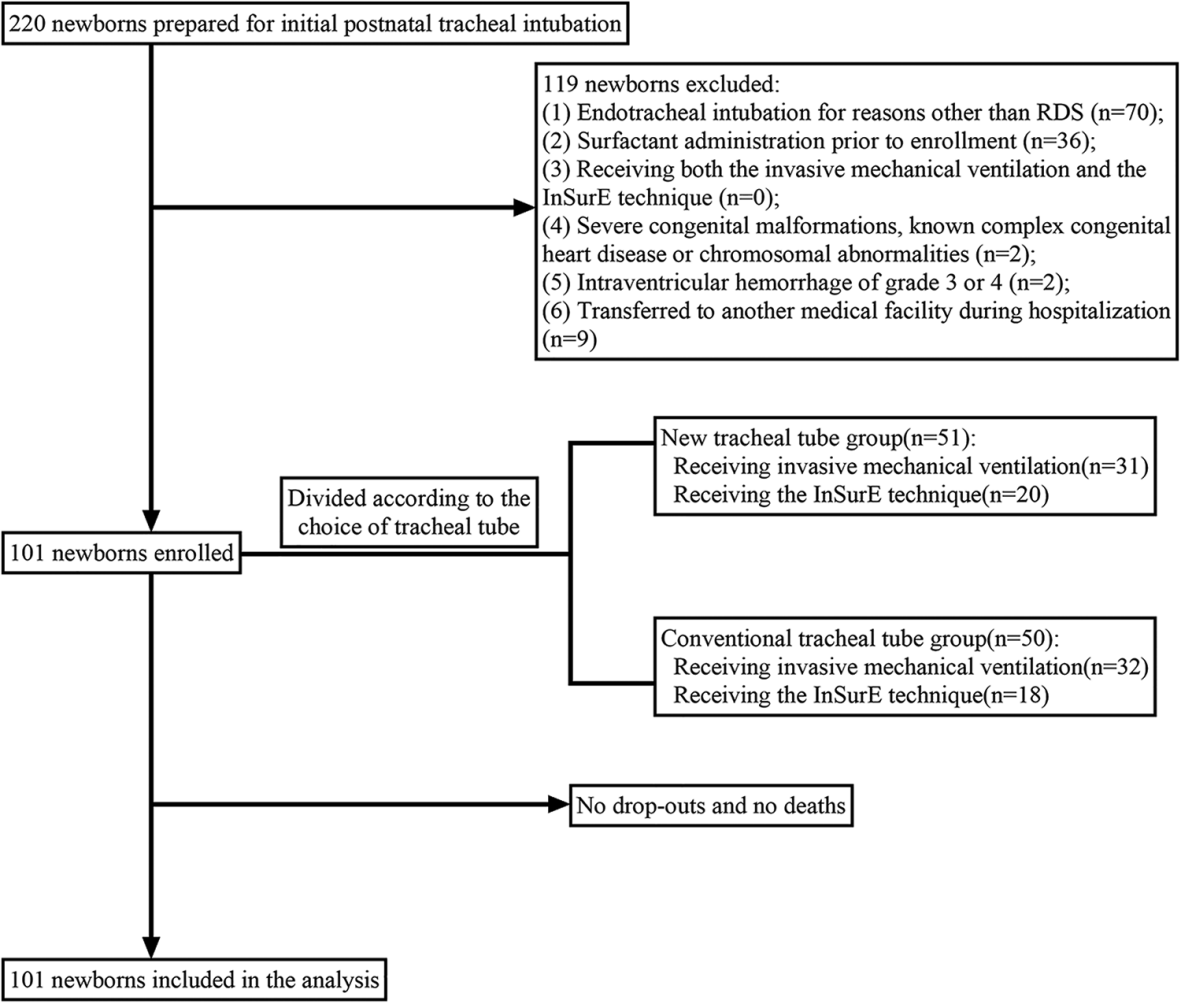
Flow diagram of participants.

A total of 101 neonates diagnosed with RDS and endotracheally intubated were eligible, of whom 63 received invasive mechanical ventilation (HFOV, SIMV, or A/C) and the remaining 38 received the InSurE technique followed by non-invasive ventilation (NCPAP, or BP-NCPAP) (Figure 2). In our study, there was no crossover between mechanical ventilation and the InSurE technique. For infants with the InSurE technique, endotracheal intubation was performed to provide access to surfactant administration, but for mechanically ventilated neonates, surfactant therapy was not mandatory. Ultimately, 78 neonates, 40 of whom were from the group with invasive mechanical ventilation, received endotracheal surfactant administration due to respiratory immaturity; for these neonates, no doses of surfactant were received by routes other than the endotracheal tube. All neonates were followed up by telephone one month after discharge. No patients dropped out or missed visits, and there were no deaths. Based on the choice of the tracheal tube, these newborns were divided into two groups: the conventional tracheal tube group (*n* = 50), and the new tracheal tube group (*n* = 51).

### Baseline characteristics of neonates and the operation of the first endotracheal intubation

Analysis of basic information on neonates and the operation of the first endotracheal intubation showed no statistical differences between the two groups, except for a significantly higher rate of surfactant administration in the new tracheal tube group than in the control group (44/51 vs. 34/50, *p* = 0.029; Table 2). Notably, the gestational age of the newborns in the new catheter group fluctuated between 28 and 41.57 weeks, with 32 of them being preterm, while the gestational age of the infants in the old catheter group ranged between 28 and 42.29 weeks, with only 23 being preterm. Stratified by gestational age (Supplementary Table S3) and the purpose of intubation (Supplementary Table S4), the data showed that for mechanically ventilated neonates (*n* = 63), the risk of surfactant application was 3.429 times higher in the new catheter group than in the old catheter group (OR = 3.429, 95% CI, 1.152–10.202, *p* = 0.024; Supplementary Table S4). Subsequently, a subgroup analysis was performed according to the purpose of endotracheal intubation (InSurE technique or invasive mechanical ventilation) (Table 3), and the data show a significantly higher percentage of preterm infants (13/31 vs. 6/32, *p* = 0.045; Table 3) and rate of surfactant administration (24/31 vs. 16/32, *p* = 0.024; Table 3) among neonates mechanically ventilated with the new tracheal tube; otherwise, there were no statistically significant differences (*p* > 0.05; Table 3). Separate stratified analyses according to gestational age (Full-term and preterm) were also performed for neonates receiving mechanical ventilation (Supplementary Table S5) and those receiving InSurE (Supplementary Table S6), and no significant association was found. Given the specificity of intratracheal administration and the fact that the response to surfactant may vary among neonates of different gestational ages (27), further subgroup analyses were performed and revealed no statistical differences in relevant indicators (*p* > 0.05; Tables 4–6).

**Table 2:** Basic information on neonates with new or conventional tracheal tubes.

	New tube; *N* = 51	Conventional tube; *N* = 50	*p*-value
Baseline characteristics of neonates			
GA, weeks	35.14[32.29–38.71]	37.36[32.86–39.18]	0.185
Premature	32/51 (62.7%)	23/50 (46.0%)	0.091
BW, g	2,200.00[1,600.00–3,000.00]	2,875.00[1,777.50–3,200.00]	0.130
Sex (male)	25/51 (49.0%)	27/50 (54.0%)	0.617
Cesarean	29/51 (56.9%)	36/50 (72.0%)	0.112
Apgar, 1 min	8.00[7.00–8.00]	7.00[4.75–8.00]	0.063
Apgar, 5 min	8.00[8.00–8.00]	8.00[7.00–8.00]	0.060
Apgar, 10 min	8.00[8.00–9.00]	8.00[8.00–9.00]	0.913
PROM	12/51 (23.5%)	11/50 (22.0%)	0.855
GDM	0/51 (0.0%)	3/50 (6.0%)	0.118
Prenatal glucocorticoid	9/51 (17.6%)	10/50 (20.0%)	0.762
Placenta praevia	2/51 (3.9%)	3/50 (6.0%)	0.678
HDCP	1/51 (2.0%)	5/50 (10.0%)	0.112
ICP	1/51 (2.0%)	0/50 (0.0%)	1.000
In vitro fertilization	4/51 (7.8%)	3/50 (6.0%)	1.000
The operation of the first endotracheal intubation			
Diameter of tube	3.00[3.00–3.50]	3.50[3.00–3.50]	0.445
Depth of tube insertion through the mouth	8.20[7.60–9.00]	8.85[7.70–9.20]	0.143
Experience of the performing physician	5.00[3.00–5.00]	4.00[4.00–6.00]	0.936
Surfactant administration *via* endotracheal tube	44/51 (86.3%)	34/50 (68.0%)	0.029*

Data are presented as mean ± SD (Range), median [IQRs], or number (%); * *P* < 0.05; GA, Gestational age; BW, Birth weight; GDM, gestational diabetes mellitus; PROM, premature rupture of the membrane; HDCP; Hypertensive disorder complicating pregnancy; ICP, intrahepatic cholestasis of pregnancy; SD, standard deviation; IQR, interquartile range.

**Table 3:** Basic information on newborns receiving invasive mechanical ventilation and the InSurE technique.

	Receiving mechanical ventilation			Receiving the InSurE technique		
	New tube; *N* = 31	Conventional tube; *N* = 32	*p*-value	New tube; *N* = 20	Conventional tube; *N* = 18	*p*-value
Baseline characteristics of neonates						
GA, weeks	37.86[33.71–40.00]	38.71[37.50–39.71]	0.289	32.59 ± 2.70 (28.00–37.57)	32.87 ± 2.46 (28.00–37.14)	0.741
Premature	13/31 (41.9%)	6/32 (18.8%)	0.045*	19/20 (95.0%)	0/18 (94.4%)	1.000
BW, g	2,700.00[2,200.00–3,250.00]	3,115.00[2,712.50–3,375.00]	0.163	1,683.50 ± 468.02 (970.00–3,000.00)	1,840.56 ± 697.31 (900.00–3,600.00)	0.416
Sex (male)	18/31 (58.1%)	17/32 (53.1%)	0.693	7/20 (35.0%)	10/18 (55.6%)	0.328
Cesarean	16/31 (51.6%)	21/32 (65.6%)	0.259	13/20 (65.0%)	15/18 (83.3%)	0.278
Apgar, 1 min	8.00[6.00–8.00]	6.87[4.00–8.00]	0.082	8.00[7.00, 8.00]	7.50[6.50, 8.00]	0.531
Apgar, 5 min	8.00[8.00–8.00]	8.00[7.00–8.00]	0.140	8.00[8.00, 8.00]	8.00[8.00, 8.00]	0.227
Apgar, 10 min	8.00[8.00–9.00]	8.00[8.00-9.00]	0.732	8.00[8.00, 8.00]	8.00[8.00, 9.00]	0.434
PROM	5/31 (16.1%)	4/32 (12.5%)	0.732	7/20 (35.0%)	7/18 (38.9%)	1.000
GDM	0/31 (0.0%)	1/32 (3.1%)	1.000	0/20 (0.0%)	2/18 (11.1%)	0.218
Prenatal glucocorticoid	2/31 (6.5%)	3/32 (9.4%)	1.000	7/20 (35.0%)	7/18 (38.9%)	1.000
Placenta praevia	0/31 (0.0%)	2/32 (6.3%)	0.492	2/20 (10.0%)	1/18 (5.6%)	1.000
HDCP	0/31 (0.0%)	2/32 (6.3%)	0.492	1/20 (5.0%)	3/18 (16.7%)	0.328
ICP	0/31 (0.0%)	0/32 (0.0%)	NA	1/20 (5.0%)	0/18 (0.0%)	1.000
In vitro fertilization	2/31 (6.5%)	0/32 (0.0%)	0.238	2/20 (10.0%)	3/18 (16.7%)	0.653
The operation of the first endotracheal intubation						
Diameter of tube	3.50[3.20–3.50]	3.50[3.50–3.50]	0.968	3.00[2.50, 3.00]	3.00[2.50, 3.00]	0.358
Depth of tube insertion through the mouth	8.70[8.20–9.20]	9.10[8.53, 9.30]	0.164	7.61 ± 0.54 (6.40–9.00)	7.79 ± 0.66 (7.00–9.60)	0.338
Experience of the performing physician	5.00[3.00–5.00]	4.00[3.00–5.00]	0.376	5.00[3.00, 5.00]	5.00[4.00, 6.00]	0.224
Surfactant administration *via* endotracheal tube	24/31 (77.4%)	16/32 (50.0%)	0.024*	20/20 (100.0%)	18/18 (100.0%)	1.000

Data are presented as mean ± SD (Range), median [IQRs], or number (%); * *P* < 0.05; GA, Gestational age; BW, Birth weight; GDM, gestational diabetes mellitus; PROM, premature rupture of the membrane; HDCP; Hypertensive disorder complicating pregnancy; ICP, intrahepatic cholestasis of pregnancy; SD, standard deviation; IQR, interquartile range.

**Table 4:** Basic information on mechanically ventilated neonates with and without surfactant administration *via* endotracheal intubation.

	With surfactant administration			Without surfactant administration		
	New tube; *N* = 24	Conventional tube; *N* = 16	*p*-value	New tube; *N* = 7	Conventional tube; *N* = 16	*p*-value
Baseline characteristics of neonates						
GA, weeks	36.42 ± 3.36 (30.86–40.86)	37.07 ± 3.40 (30.14–41.14)	0.553	38.79 ± 2.55 (33.71–41.57)	39.06 ± 1.22 (37.29–42.29)	0.733
Premature	12/24 (50.0%)	6/16 (37.5%)	0.436	1/7 (14.3%)	0/16 (0.0%)	0.304
BW, g	2,678.75 ± 776.39 (1,500.00–4,200.00)	2,766.88 ± 719.23 (1,470.00–3,800.00)	0.719	2,835.71 ± 808.49 (1,200.00–3,760.00)	3,126.25 ± 396.77 (2,300.00–3,600.00)	0.254
Sex (male)	14/24 (58.3%)	8/16 (50.0%)	0.604	4/7 (57.1%)	9/16 (56.3%)	1.000
Cesarean	11/24 (45.8%)	12/16 (75.0%)	0.068	5/7 (71.4%)	9/16 (56.3%)	0.657
Apgar, 1 min	8.00[5.25, 8.00]	8.00[6.00, 8.00]	0.747	8.00[6.00, 8.00]	5.00[2.25, 7.75]	0.096
Apgar, 5 min	8.00[7.25, 8.00]	8.00[7.17, 8.00]	0.770	8.14 ± 0.69 (7.00–9.00)	7.15 ± 1.62 (4.00–10.00)	0.137
Apgar, 10 min	8.00[8.00, 9.00]	8.00[8.00, 9.00]	0.414	9.00[9.00, 9.00]	8.00[7.25, 9.00]	0.076
PROM	5/24 (20.8%)	1/16 (6.3%)	0.373	0/7 (0.0%)	3/16 (18.8%)	0.526
GDM	0/24 (0.0%)	1/16 (6.3%)	0.400	0/7 (0.0%)	0/16 (0.0%)	1.000
Prenatal glucocorticoid	2/24 (8.3%)	3/16 (18.8%)	0.373	0/7 (0.0%)	0/16 (0.0%)	1.000
Placenta praevia	0/24 (0.0%)	1/16 (6.3%)	0.400	0/7 (0.0%)	1/16 (6.3%)	1.000
HDCP	0/24 (0.0%)	2/16 (12.5%)	0.154	0/7 (0.0%)	0/16 (0.0%)	1.000
ICP	0/24 (0.0%)	0/16 (0.0%)	1.000	0/7 (0.0%)	0/16 (0.0%)	1.000
In vitro fertilization	1/24 (4.2%)	0/16 (0.0%)	1.000	1/7 (14.3%)	0/16 (0.0%)	0.304
The operation of the first endotracheal intubation						
Diameter of tube	3.50[3.20, 3.50]	3.50[3.13, 3.50]	0.831	3.50[3.00, 4.00]	3.50[3.50, 3.50]	1.000
Depth of tube insertion through the mouth	8.65[7.90, 9.20]	9.00[8.00, 9.20]	0.524	9.00[8.60, 9.30]	9.20[9.00, 9.45]	0.480
Experience of the performing physician	5.00[3.00, 5.00]	4.00[3.25, 5.75]	0.719	5.00[5.00, 6.00]	4.00[3.00, 5.00]	0.180
Surfactant administration *via* endotracheal tube	24/24 (100.0%)	16/16 (100.0%)	1.000	0/7 (0.0%)	0/16 (0.0%)	1.000

Data are presented as mean ± SD (Range), median [IQRs], or number (%); GA, Gestational age; BW, Birth weight; GDM, gestational diabetes mellitus; PROM, premature rupture of the membrane; HDCP; Hypertensive disorder complicating pregnancy; ICP, intrahepatic cholestasis of pregnancy; SD, standard deviation; IQR, interquartile range.

**Table 5:** Basic information on preterm and term neonates requiring surfactant administration *via* endotracheal tube during mechanical ventilation.

	Preterm neonates			Term neonates		
	New tube; *N* = 12	Conventional tube; *N* = 6	*p*-value	New tube; *N* = 12	Conventional tube; *N* = 10	*p*-value
Baseline characteristics of neonates						
GA, weeks	33.52 ± 1.85 (30.86–36.57)	33.43 ± 2.57 (30.14–36.86)	0.929	39.31 ± 1.40 (37.00–40.86)	39.26 ± 1.21 (37.71–41.14)	0.925
BW, g	2,125.83 ± 559.54 (1,500.00–3,250.00)	2,138.33 ± 651.93 (1,470.00–3,000.00)	0.967	3,231.67 ± 529.41 (2,470.00–4,200.00)	3,144.00 ± 452.36 (2,080.00–3,800.00)	0.684
Sex (male)	7/12 (58.3%)	4/6 (66.7%)	1.000	7/12 (58.3%)	4/10 (40.0%)	0.670
Cesarean	7/12 (58.3%)	5/6 (83.3%)	0.600	4/12 (33.3%)	7/10 (70.0%)	0.198
Apgar, 1 min	7.42 ± 1.83 (4.00–10.00)	6.00 ± 1.67 (4.00–8.00)	0.132	8.00[4.25, 8.00]	8.00[7.50, 8.00]	0.220
Apgar, 5 min	8.00[8.00, 8.00]	7.50[6.00, 8.00]	0.146	8.00[7.00, 8.00]	8.00[7.92, 8.00]	0.399
Apgar, 10 min	8.00[8.00, 8.25]	8.00[8.00, 8.06]	0.405	8.00[7.25, 9.00]	8.63[8.00, 9.00]	0.233
PROM	2/12 (16.7%)	1/6 (16.7%)	1.000	3/12 (25.0%)	0/10 (0.0%)	0.221
GDM	0/12 (0.0%)	1/6 (16.7%)	0.333	0/12 (0.0%)	0/10 (0.0%)	1.000
Prenatal glucocorticoid	2/12 (16.7%)	3/6 (50.0%)	0.268	0/12 (0.0%)	0/10 (0.0%)	1.000
Placenta praevia	0/12 (0.0%)	1/6 (16.7%)	0.333	0/12 (0.0%)	0/10 (0.0%)	1.000
HDCP	0/12 (0.0%)	2/6 (33.3%)	0.098	0/12 (0.0%)	0/10 (0.0%)	1.000
ICP	0/12 (0.0%)	0/6 (0.0%)	1.000	0/12 (0.0%)	0/10 (0.0%)	1.000
In vitro fertilization	1/12 (8.3%)	0/6 (0.0%)	1.000	0/12 (0.0%)	0/10 (0.0%)	1.000
The operation of the first endotracheal intubation						
Diameter of tube	3.35[3.00, 3.50]	3.00[3.00, 3.50]	0.537	3.50[3.50, 4.00]	3.50[3.50, 3.63]	0.482
Depth of tube insertion through the mouth	8.10 ± 0.56 (7.50–9.20)	8.1333 ± 0.65 (7.50–9.00)	0.912	9.20 ± 0.55 (8.40–10.20)	9.14 ± 0.48 (8.00–9.80)	0.789
Experience of the performing physician	5.00[3.50, 5.00]	5.50[3.50, 6.25]	0.279	4.50[3.00, 5.00]	4.00[3.00, 4.00]	0.557
Surfactant administration *via* endotracheal tube	12/12 (100.0%)	6/6 (100.0%)	1.000	12/12 (100.0%)	10/10 (100.0%)	1.000

Data are presented as mean ± SD (Range), median [IQRs], or number (%); GA, Gestational age; BW, Birth weight; GDM, gestational diabetes mellitus; PROM, premature rupture of the membrane; HDCP; Hypertensive disorder complicating pregnancy; ICP, intrahepatic cholestasis of pregnancy; SD, standard deviation; IQR, interquartile range.

**Table 6:** Basic information on premature newborns with gestational age ≤32 weeks and >32 weeks, receiving the inSurE technique.

	Premature newborns with gestational age ≤32 weeks			Premature newborns with gestational age >32 weeks		
	New tube; *N* = 9	Conventional tube; *N* = 5	*p*-value	New tube; *N* = 10	Conventional tube; *N* = 12	*p*-value
Baseline characteristics of neonates						
GA, weeks	30.30 ± 1.33 (28.00–31.86)	29.91 ± 1.50 (28.00–31.57)	0.626	33.93[32.50, 36.04]	32.86[32.75, 35.03]	0.947
BW, g	1,366.67 ± 261.20 (970.00–1,750.00)	1,176.00 ± 279.07 (900.00–1,600.00)	0.225	1,837.00 ± 287.48 (1,490.00–2,310.00)	1,970.83 ± 471.66 (1,320.00–2,850.00)	0.443
Sex (male)	3/9 (33.3%)	3/5 (60.0%)	0.580	4/10 (40.0%)	6/12 (50.0%)	0.691
Cesarean	4/9 (44.4%)	4/5 (80.0%)	0.301	8/10 (80.0%)	10/12 (83.3%)	1.000
Apgar, 1 min	8.00[6.50, 8.00]	7.00[4.50, 8.00]	0.352	7.50[7.00, 8.00]	7.50[7.00, 8.00]	0.828
Apgar, 5 min	8.00[8.00, 8.00]	8.00[7.50, 8.00]	0.661	8.00[8.00, 8.00]	8.00[8.00, 8.00]	0.186
Apgar, 10 min	8.00[8.00, 8.50]	8.00[8.00, 8.00]	0.642	8.00[8.00, 8.00]	8.00[8.00, 9.00]	0.204
PROM	3/9 (33.3%)	2/5 (40.0%)	1.000	4/10 (40.0%)	5/12 (41.7%)	1.000
GDM	0/9 (0.0%)	2/5 (40.0%)	0.110	0/10 (0.0%)	0/12 (0.0%)	1.000
Prenatal glucocorticoid	5/9 (55.6%)	4/5 (80.0%)	0.580	2/10 (20.0%)	3/12 (25.0%)	1.000
Placenta praevia	2/9 (22.2%)	0/5 (0.0%)	0.505	0/10 (0.0%)	1/12 (8.3%)	1.000
HDCP	0/9 (0.0%)	1/5 (20.0%)	0.357	1/10 (10.0%)	2/12 (16.7%)	1.000
ICP	0/9 (0.0%)	0/5 (0.0%)	1.000	1/10 (10.0%)	0/12 (0.0%)	0.455
In vitro fertilization	0/9 (0.0%)	0/5 (0.0%)	1.000	2/10 (20.0%)	3/12 (25.0%)	1.000
The operation of the first endotracheal intubation						
Diameter of tube	2.50[2.50, 3.00]	3.00[2.50, 3.00]	0.591	3.00[2.50, 3.00]	3.00[2.63, 3.00]	0.599
Depth of tube insertion through the mouth	7.30[7.00, 7.60]	7.00[7.00, 7.55]	0.631	7.79 ± 0.27 (7.50–8.30)	7.88 ± 0.43 (7.30–8.80)	0.561
Experience of the performing physician	4.22 ± 1.79 (2.00–8.00)	4.20 ± 1.10 (3.00–6.00)	0.980	5.00[3.00, 5.00]	5.00[4.00, 6.00]	0.271
Surfactant administration *via* endotracheal tube	9/9 (100.0%)	5/5 (100.0%)	1.000	10/10 (100.0%)	12/12 (100.0%)	1.000

Data are presented as mean ± SD (Range), median [IQRs], or number (%); GA, Gestational age; BW, Birth weight; GDM, gestational diabetes mellitus; PROM, premature rupture of the membrane; HDCP; Hypertensive disorder complicating pregnancy; ICP, intrahepatic cholestasis of pregnancy; SD, standard deviation; IQR, interquartile range.

### Safety

No statistical differences were found between the two groups in terms of available safety outcomes, either during (0/51 vs. 0/50, *p* = 1.000; Table 7) or after (2/51 vs. 3/50, *p* = 0.678; Table 7) intubation, even after subgroup analyses based on the reason for intubation (*p* > 0.05; Table 8), whether the drug was administered endotracheally (*p* > 0.05; Table 9), and gestational age (*p* > 0.05; Tables 10, 11). Similarly, no significant association was found between the application of the novel double-lumen tracheal tube and safety outcome indicators after controlling for the confounding factor of gestational age or the purpose of intubation. (*p* > 0.05; Supplementary Tables S7–10).

**Table 7:** Clinical outcomes of neonates with the new or conventional tracheal tubes.

	New tube; *N* = 51	Conventional tube; *N* = 50	*p*-value
Adverse reactions during intubation			
Total	0/51 (0.0%)	0/50 (0.0%)	1.000
Oral mucosal injury	0/51 (0.0%)	0/50 (0.0%)	1.000
Bleeding airway injury	0/51 (0.0%)	0/50 (0.0%)	1.000
Respiratory depression	0/51 (0.0%)	0/50 (0.0%)	1.000
Bradycardia	0/51 (0.0%)	0/50 (0.0%)	1.000
Tachycardia	0/51 (0.0%)	0/50 (0.0%)	1.000
Arterial hypotension	0/51 (0.0%)	0/50 (0.0%)	1.000
Laryngospasm	0/51 (0.0%)	0/50 (0.0%)	1.000
Blockage of the drug delivery tube	0/51 (0.0%)	0/50 (0.0%)	1.000
Distortion of the dominant tube	0/51 (0.0%)	0/50 (0.0%)	1.000
Adverse reactions after intubation			
Total	2/51 (3.9%%)	3/50 (6.0%)	0.678
Coughing and wheezing	0/51 (0.0%)	0/50 (0.0%)	1.000
Recurrent choking on milk	0/51 (0.0%)	0/50 (0.0%)	1.000
Pulmonary hemorrhage	0/51 (0.0%)	0/50 (0.0%)	1.000
Emphysema	0/51 (0.0%)	0/50 (0.0%)	1.000
IVH grade 3 or 4	0/51 (0.0%)	1/50 (2.0%)	0.495
Secondary infection	1/51 (2.0%)	3/50 (6.0%)	0.362
Pneumothorax	1/51 (2.0%)	0/50 (0.0%)	1.000
Concurrent laryngospasm	0/51 (0.0%)	0/50 (0.0%)	1.000
Efficacy of tracheal tubes			
Total number of intubations	1.00[1.00–1.00]	1.00[1.00–1.00]	0.977
Duration of MV (hours)	63.00[0.00–115.00]	88.50[0.00–120.50]	0.358
Duration of non- invasive ventilation (hours)	89.00[48.00–140.00]	81.00[48.00–144.00]	0.878
Duration of total ventilation (hours)	152.00[89.00–240.00]	150.50[108.75–288.00]	0.419
Duration of oxygen therapy (days)	5.00[2.00–19.00]	6.00[3.00–18.00]	0.718
Length of hospital stay (days)	22.00[14.00–32.00]	20.50[13.75–30.00]	0.514
Discharged with oxygen	20/51 (39.2%)	16/50 (32.0%)	0.449
Recurrent dyspnea one month after discharge	0/51 (0.0%)	0/50 (0.0%)	1.000

Data are presented as mean ± SD (Range), median [IQRs], or number (%); IVH, Intraventricular hemorrhage; SD, standard deviation; IQR, interquartile range.

**Table 8:** Clinical outcomes of neonates receiving invasive mechanical ventilation and the inSurE technique.

	Receiving mechanical ventilation			Receiving the InSurE technique		
	New tube; *N* = 31	Conventional tube; *N* = 32	*p*-value	New tube; *N* = 20	Conventional tube; *N* = 18	*p*-value
Adverse reactions during intubation						
Total	0/31 (0.0%)	0/32 (0.0%)	1.000	0/20 (0.0%)	0/18 (0.0%)	1.000
Oral mucosal injury	0/31 (0.0%)	0/32 (0.0%)	1.000	0/20 (0.0%)	0/18 (0.0%)	1.000
Bleeding airway injury	0/31 (0.0%)	0/32 (0.0%)	1.000	0/20 (0.0%)	0/18 (0.0%)	1.000
Respiratory depression	0/31 (0.0%)	0/32 (0.0%)	1.000	0/20 (0.0%)	0/18 (0.0%)	1.000
Bradycardia	0/31 (0.0%)	0/32 (0.0%)	1.000	0/20 (0.0%)	0/18 (0.0%)	1.000
Tachycardia	0/31 (0.0%)	0/32 (0.0%)	1.000	0/20 (0.0%)	0/18 (0.0%)	1.000
Arterial hypotension	0/31 (0.0%)	0/32 (0.0%)	1.000	0/20 (0.0%)	0/18 (0.0%)	1.000
Laryngospasm	0/31 (0.0%)	0/32 (0.0%)	1.000	0/20 (0.0%)	0/18 (0.0%)	1.000
Blockage of the drug delivery tube	0/31 (0.0%)	0/32 (0.0%)	1.000	0/20 (0.0%)	0/18 (0.0%)	1.000
Distortion of the dominant tube	0/31 (0.0%)	0/32 (0.0%)	1.000	0/20 (0.0%)	0/18 (0.0%)	1.000
Adverse reactions after intubation						
Total	1/31 (3.2%)	3/32 (9.4%)	0.613	1/20 (5.0%)	0/18 (0.0%)	1.000
Coughing and wheezing	0/31 (0.0%)	0/32 (0.0%)	1.000	0/20 (0.0%)	0/18 (0.0%)	1.000
Recurrent choking on milk	0/31 (0.0%)	0/32 (0.0%)	1.000	0/20 (0.0%)	0/18 (0.0%)	1.000
Pulmonary hemorrhage	0/31 (0.0%)	0/32 (0.0%)	1.000	0/20 (0.0%)	0/18 (0.0%)	1.000
Emphysema	0/31 (0.0%)	0/32 (0.0%)	1.000	0/20 (0.0%)	0/18 (0.0%)	1.000
IVH grade 3 or 4	0/31 (0.0%)	1/32 (3.1%)	1.000	0/20 (0.0%)	0/18 (0.0%)	1.000
Secondary infection	0/31 (0.0%)	3/32 (9.4%)	0.238	1/20 (5.0%)	0/18 (0.0%)	1.000
Pneumothorax	1/31 (3.2%)	0/32 (0.0%)	0.492	0/20 (0.0%)	0/18 (0.0%)	1.000
Concurrent laryngospasm	0/31 (0.0%)	0/32 (0.0%)	1.000	0/20 (0.0%)	0/18 (0.0%)	1.000
Efficacy of tracheal tubes						
Total number of intubations	1.00[1.00, 1.00]	1.00[1.00, 1.00]	0.670	1.00[1.00, 1.00]	1.00[1.00, 1.00]	0.617
Duration of MV (hours)	103.32 ± 48.20 (20.00–213.00)	121.91 ± 58.33 (24.00–240.00)	0.174	/	/	/
Duration of non-invasive ventilation (hours)	72.00[48.00, 120.00]	72.00[48.00, 120.00]	0.847	92.50[62.00, 153.75]	108.00[66.00, 200.25]	0.884
Duration of total ventilation (hours)	193.52 ± 85.74 (20.00–384.00)	222.25 ± 118.27 (24.00–564.00)	0.275	92.50[62.00, 153.75]	108.00[66.00, 200.25]	0.884
Duration of oxygen therapy (days)	4.00[2.00, 10.00]	4.00[3.00, 9.75]	0.519	19.30 ± 14.56 (0.00–44.00)	18.78 ± 14.03 (2.00–50.00)	0.911
Length of hospital stay (days)	17.00[14.00, 27.00]	16.00[13.00, 22.75]	0.577	32.40 ± 16.11 (7.00–68.00)	32.39 ± 18.61 (9.00–74.00)	0.998
Discharged with oxygen	8/31 (25.8%)	7/32 (21.9%)	0.714	12/20 (60.0%)	9/18 (50.0%)	0.745
Recurrent dyspnea one month after discharge	0/31 (0.0%)	0/32 (0.0%)	1.000	0/20 (0.0%)	0/18 (0.0%)	1.000

Data are presented as mean ± SD (Range), median [IQRs], or number (%); IVH, Intraventricular hemorrhage; SD, standard deviation; IQR, interquartile range.

**Table 9:** Clinical outcomes of mechanically ventilated neonates with and without surfactant administration *via* endotracheal intubation.

	With surfactant administration			Without surfactant administration		
	New tube; *N* = 24	Conventional tube; *N* = 16	*p*-value	New tube; *N* = 7	Conventional tube; *N* = 16	*p*-value
Adverse reactions during intubation						
Total	0/24 (0.0%)	0/16 (0.0%)	1.000	0/7 (0.0%)	0/16 (0.0%)	1.000
Drug reflux	0/24 (0.0%)	0/16 (0.0%)	1.000	0/7 (0.0%)	0/16 (0.0%)	1.000
Oral mucosal injury	0/24 (0.0%)	0/16 (0.0%)	1.000	0/7 (0.0%)	0/16 (0.0%)	1.000
Bleeding airway injury	0/24 (0.0%)	0/16 (0.0%)	1.000	0/7 (0.0%)	0/16 (0.0%)	1.000
Respiratory depression	0/24 (0.0%)	0/16 (0.0%)	1.000	0/7 (0.0%)	0/16 (0.0%)	1.000
Bradycardia	0/24 (0.0%)	0/16 (0.0%)	1.000	0/7 (0.0%)	0/16 (0.0%)	1.000
Tachycardia	0/24 (0.0%)	0/16 (0.0%)	1.000	0/7 (0.0%)	0/16 (0.0%)	1.000
Arterial hypotension	0/24 (0.0%)	0/16 (0.0%)	1.000	0/7 (0.0%)	0/16 (0.0%)	1.000
Laryngospasm	0/24 (0.0%)	0/16 (0.0%)	1.000	0/7 (0.0%)	0/16 (0.0%)	1.000
Blockage of the drug delivery tube	0/24 (0.0%)	0/16 (0.0%)	1.000	0/7 (0.0%)	0/16 (0.0%)	1.000
Distortion of the dominant tube	0/24 (0.0%)	0/16 (0.0%)	1.000	0/7 (0.0%)	0/16 (0.0%)	1.000
Adverse reactions after intubation						
Total	1/24 (4.2%)	3/16 (18.8%)	0.283	0/7 (0.0%)	0/16 (0.0%)	1.000
Coughing and wheezing	0/24 (0.0%)	0/16 (0.0%)	1.000	0/7 (0.0%)	0/16 (0.0%)	1.000
Recurrent choking on milk	0/24 (0.0%)	0/16 (0.0%)	1.000	0/7 (0.0%)	0/16 (0.0%)	1.000
Pulmonary hemorrhage	0/24 (0.0%)	0/16 (0.0%)	1.000	0/7 (0.0%)	0/16 (0.0%)	1.000
Emphysema	0/24 (0.0%)	0/16 (0.0%)	1.000	0/7 (0.0%)	0/16 (0.0%)	1.000
IVH grade 3 or 4	0/24 (0.0%)	1/16 (6.3%)	0.400	0/7 (0.0%)	0/16 (0.0%)	1.000
Secondary infection	0/24 (0.0%)	3/16 (18.8%)	0.057	0/7 (0.0%)	0/16 (0.0%)	1.000
Pneumothorax	1/24 (4.2%)	0/16 (0.0%)	1.000	0/7 (0.0%)	0/16 (0.0%)	1.000
Concurrent laryngospasm	0/24 (0.0%)	0/16 (0.0%)	1.000	0/7 (0.0%)	0/16 (0.0%)	1.000
Efficacy of tracheal tubes						
Total number of intubations	1.00[1.00, 1.00]	1.00[1.00, 1.00]	0.333	1.00[1.00, 1.00]	1.00[1.00, 1.00]	0.538
Duration of MV (hours)	96.50[74.00, 144.00]	121.00[96.00, 196.50]	0.037*	84.00 ± 54.20 (20.00–178.00)	99.50 ± 55.10 (24.00–216.00)	0.540
Duration of non-invasive ventilation (hours)	96.00[48.00, 120.00]	108.00[54.00, 108.00]	0.311	71.00[45.00, 96.00]	72.00[48.00, 90.00]	0.813
Duration of total ventilation (hours)	205.71 ± 80.24 (65.00–384.00)	277.56 ± 117.84 (122.00–564.00)	0.027*	151.71 ± 97.15 (20.00–322.00)	166.94 ± 92.18 (24.00–409.00)	0.723
Duration of oxygen therapy (days)	4.00[2.00, 9.50]	4.50[3.00, 14.00]	0.279	3.00[2.00, 37.00]	3.50[2.25, 5.75]	0.839
Length of hospital stay (days)	20.50 ± 6.88 (11.00–37.00)	22.31 ± 9.45 (10.00–39.00)	0.486	13.00[9.00, 33.00]	14.50[13.00, 19.00]	0.521
Discharged with oxygen	6/24 (25.0%)	4/16 (25.0%)	1.000	2/7 (28.6%)	3/16 (18.8%)	0.621
Recurrent dyspnea one month after discharge	0/24 (0.0%)	0/16 (0.0%)	1.000	0/7 (0.0%)	0/16 (0.0%)	1.000

Data are presented as mean ± SD (Range), median [IQRs], or number (%); * *P* < 0.05; IVH, Intraventricular hemorrhage; SD, standard deviation; IQR, interquartile range.

**Table 10:** Clinical outcomes of preterm and term neonates requiring surfactant administration *via* endotracheal tube during mechanical ventilation.

	Preterm neonates			Term neonates		
	New tube; *N* = 12	Conventional tube; *N* = 6	*p*-value	New tube; *N* = 12	Conventional tube; *N* = 10	*p*-value
Adverse reactions during intubation						
Total	0/12 (0.0%)	0/6 (0.0%)	1.000	0/12 (0.0%)	0/10 (0.0%)	1.000
Drug reflux	0/12 (0.0%)	0/6 (0.0%)	1.000	0/12 (0.0%)	0/10 (0.0%)	1.000
Oral mucosal injury	0/12 (0.0%)	0/6 (0.0%)	1.000	0/12 (0.0%)	0/10 (0.0%)	1.000
Bleeding airway injury	0/12 (0.0%)	0/6 (0.0%)	1.000	0/12 (0.0%)	0/10 (0.0%)	1.000
Respiratory depression	0/12 (0.0%)	0/6 (0.0%)	1.000	0/12 (0.0%)	0/10 (0.0%)	1.000
Bradycardia	0/12 (0.0%)	0/6 (0.0%)	1.000	0/12 (0.0%)	0/10 (0.0%)	1.000
Tachycardia	0/12 (0.0%)	0/6 (0.0%)	1.000	0/12 (0.0%)	0/10 (0.0%)	1.000
Arterial hypotension	0/12 (0.0%)	0/6 (0.0%)	1.000	0/12 (0.0%)	0/10 (0.0%)	1.000
Laryngospasm	0/12 (0.0%)	0/6 (0.0%)	1.000	0/12 (0.0%)	0/10 (0.0%)	1.000
Blockage of the drug delivery tube	0/12 (0.0%)	0/6 (0.0%)	1.000	0/12 (0.0%)	0/10 (0.0%)	1.000
Distortion of the dominant tube	0/12 (0.0%)	0/6 (0.0%)	1.000	0/12 (0.0%)	0/10 (0.0%)	1.000
Adverse reactions after intubation						
Total	0/12 (0.0%)	1/6 (16.7%)	0.333	1/12 (8.3%)	2/10 (20.0%)	0.571
Coughing and wheezing	0/12 (0.0%)	0/6 (0.0%)	1.000	0/12 (0.0%)	0/10 (0.0%)	1.000
Recurrent choking on milk	0/12 (0.0%)	0/6 (0.0%)	1.000	0/12 (0.0%)	0/10 (0.0%)	1.000
Pulmonary hemorrhage	0/12 (0.0%)	0/6 (0.0%)	1.000	0/12 (0.0%)	0/10 (0.0%)	1.000
Emphysema	0/12 (0.0%)	0/6 (0.0%)	1.000	0/12 (0.0%)	0/10 (0.0%)	1.000
IVH grade 3 or 4	0/12 (0.0%)	1/6 (16.7%)	0.333	0/12 (0.0%)	0/10 (0.0%)	1.000
Secondary infection	0/12 (0.0%)	1/6 (16.7%)	0.333	0/12 (0.0%)	2/10 (20.0%)	0.195
Pneumothorax	0/12 (0.0%)	0/6 (0.0%)	1.000	1/12 (8.3%)	0/10 (0.0%)	1.000
Concurrent laryngospasm	0/12 (0.0%)	0/6 (0.0%)	1.000	0/12 (0.0%)	0/10 (0.0%)	1.000
Efficacy of tracheal tubes						
Total number of intubations	1.00[1.00, 1.00]	1.00[1.00, 1.25]	0.606	1.00[1.00, 1.00]	1.00[1.00, 1.00]	0.273
Duration of MV (hours)	101.75 ± 39.72 (36.00–168.00)	155.50 ± 51.49 (96.00–240.00)	0.026*	120.00[64.00, 155.25]	114.50[96.00, 206.25]	0.529
Duration of non-invasive ventilation (hours)	114.25 ± 70.96 (24.00–240.00)	196.00 ± 135.27 (72.00–456.00)	0.107	79.25 ± 35.36 (25.00–120.00)	95.60 ± 79.21 (0.00–288.00)	0.527
Duration of total ventilation (hours)	216.00 ± 81.60 (96.00–384.00)	351.50 ± 113.79 (240.00–564.00)	0.010*	195.42 ± 81.08 (65.00–330.00)	233.20 ± 100.60 (122.00–408.00)	0.341
Duration of oxygen therapy (days)	9.75 ± 6.02 (1.00–19.00)	17.33 ± 8.43 (5.00–19.00)	0.042*	2.00[2.00, 3.75]	3.00[2.00, 5.25]	0.175
Length of hospital stay (days)	24.42 ± 6.87 (14.00–37.00)	30.50 ± 8.80 (18.00–39.00)	0.126	16.58 ± 4.25 (11.00–26.00)	17.40 ± 5.85 (10.00–29.00)	0.709
Discharged with oxygen	3/12 (25.0%)	3/6 (50.0%)	0.344	3/12 (25.0%)	1/10 (10.0%)	0.594
Recurrent dyspnea one month after discharge	0/12 (0.0%)	0/6 (0.0%)	1.000	0/12 (0.0%)	0/10 (0.0%)	1.000

Data are presented as mean ± SD (Range), median [IQRs], or number (%); * *P* < 0.05; IVH, Intraventricular hemorrhage; SD, standard deviation; IQR, interquartile range.

**Table 11:** Clinical outcomes of premature newborns receiving the inSurE technique with gestational age ≤32 weeks and >32 weeks.

	Premature newborns with gestational age ≤32 weeks			Premature newborns with gestational age >32 weeks		
	New tube; *N* = 9	Conventional tube; *N* = 5	*p*-value	New tube; *N* = 10	Conventional tube; *N* = 12	*p*-value
Adverse reactions during intubation						
Total	0/9 (0.0%)	0/5 (0.0%)	1.000	0/10 (0.0%)	0/12 (0.0%)	1.000
Drug reflux	0/9 (0.0%)	0/5 (0.0%)	1.000	0/10 (0.0%)	0/12 (0.0%)	1.000
Oral mucosal injury	0/9 (0.0%)	0/5 (0.0%)	1.000	0/10 (0.0%)	0/12 (0.0%)	1.000
Bleeding airway injury	0/9 (0.0%)	0/5 (0.0%)	1.000	0/10 (0.0%)	0/12 (0.0%)	1.000
Respiratory depression	0/9 (0.0%)	0/5 (0.0%)	1.000	0/10 (0.0%)	0/12 (0.0%)	1.000
Bradycardia	0/9 (0.0%)	0/5 (0.0%)	1.000	0/10 (0.0%)	0/12 (0.0%)	1.000
Tachycardia	0/9 (0.0%)	0/5 (0.0%)	1.000	0/10 (0.0%)	0/12 (0.0%)	1.000
Arterial hypotension	0/9 (0.0%)	0/5 (0.0%)	1.000	0/10 (0.0%)	0/12 (0.0%)	1.000
Laryngospasm	0/9 (0.0%)	0/5 (0.0%)	1.000	0/10 (0.0%)	0/12 (0.0%)	1.000
Blockage of the drug delivery tube	0/9 (0.0%)	0/5 (0.0%)	1.000	0/10 (0.0%)	0/12 (0.0%)	1.000
Distortion of the dominant tube	0/9 (0.0%)	0/5 (0.0%)	1.000	0/10 (0.0%)	0/12 (0.0%)	1.000
Adverse reactions after intubation						
Total	1/9 (11.1%)	0/5 (0.0%)	1.000	0/10 (0.0%)	0/12 (0.0%)	1.000
Coughing and wheezing	0/9 (0.0%)	0/5 (0.0%)	1.000	0/10 (0.0%)	0/12 (0.0%)	1.000
Recurrent choking on milk	0/9 (0.0%)	0/5 (0.0%)	1.000	0/10 (0.0%)	0/12 (0.0%)	1.000
Pulmonary hemorrhage	0/9 (0.0%)	0/5 (0.0%)	1.000	0/10 (0.0%)	0/12 (0.0%)	1.000
Emphysema	0/9 (0.0%)	0/5 (0.0%)	1.000	0/10 (0.0%)	0/12 (0.0%)	1.000
IVH grade 3 or 4	0/9 (0.0%)	0/5 (0.0%)	1.000	0/10 (0.0%)	0/12 (0.0%)	1.000
Secondary infection	1/9 (11.1%)	0/5 (0.0%)	1.000	0/10 (0.0%)	0/12 (0.0%)	1.000
Pneumothorax	0/9 (0.0%)	0/5 (0.0%)	1.000	0/10 (0.0%)	0/12 (0.0%)	1.000
Concurrent laryngospasm	0/9 (0.0%)	0/5 (0.0%)	1.000	0/10 (0.0%)	0/12 (0.0%)	1.000
Efficacy of tracheal tubes						
Total number of intubations	1.00[1.00, 1.50]	1.00[1.00, 1.50]	0.925	1.00[1.00, 1.00]	1.00[1.00, 1.00]	1.000
Duration of non-invasive ventilation (hours)	156.00[112.00, 432.00]	528.00[56.50, 1,116.00]	0.505	68.50[48.00, 95.75]	100.50[72.00, 138.00]	0.164
Duration of oxygen therapy (days)	26.22 ± 13.75 (0.00–44.00)	30.40 ± 16.88 (12.00–50.00)	0.624	8.00[3.75, 30.25]	15.50[6.25, 23.75]	0.817
Length of hospital stay (days)	43.89 ± 13.89 (28.00–68.00)	54.40 ± 18.53 (30.00–74.00)	0.250	24.60 ± 10.27 (12.00–37.00)	25.17 ± 9.15 (12.00–41.00)	0.893
Discharged with oxygen	7/9 (77.8%)	5/5 (100.0%)	0.505	5/10 (50.0%)	4/12 (33.3%)	0.666
Recurrent dyspnea one month after discharge	0/9 (0.0%)	0/5 (0.0%)	1.000	0/10 (0.0%)	0/12 (0.0%)	1.000

Data are presented as mean ± SD (Range), median [IQRs], or number (%); IVH, Intraventricular hemorrhage; SD, standard deviation; IQR, interquartile range.

### Efficacy

The effectiveness of the new tracheal tube was evaluated using a conventional tracheal tube as a control, and the data showed no significant differences in all relevant indicators between the two groups of infants (*p* > 0.05; Table 7). A stratified analysis of the indicator “Discharged with oxygen” was performed according to gestational age (*p* = 0.856; Supplementary Table 11) and purpose of intubation (*p* = 0.493; Supplementary Table 11), and the data showed no significant association between the application of the new tracheal tube and the clinical outcome; for other outcome indicators targeting effectiveness, the stratified analysis was not applicable. Considering the limitations of the stratified analysis and the heterogeneity among study subjects, we performed some detailed subgroup analyses based on the above criteria (Tables 8–11).

For mechanically ventilated infants requiring surfactant administration (*n* = 40; GA fluctuating between 30.14 and 41.14 weeks), the application of the new tracheal tube resulted in a significant reduction in the duration of mechanical ventilation (96.50[74.00, 144.00] vs. 121.00[96.00, 196.50] hours, *p* = 0.037; Table 9) and total ventilation (205.71 ± 80.24 vs. 277.56 ± 117.84 h, *p* = 0.027; Table 9). However, there were no statistical differences between the two groups in terms of the number of intubations (1.00[1.00, 1.00] vs. 1.00[1.00, 1.00], *p* = 0.333; Table 9), duration of non-invasive ventilation (96.00[48.00, 120.00] vs. 108.00[54.00, 108.00] hours, *p* = 0.311; Table 9), duration of oxygen therapy (4.00[2.00, 9.50] vs. 4.50[3.00, 14.00] days, *p* = 0.279; Table 9), length of stay (20.50 ± 6.88 vs. 22.31 ± 9.45 days, *p* = 0.486; Table 9), rate of discharge with oxygen (6/24 vs. 4/16, *p* = 1.000; Table 9), and rate of recurrent dyspnea one month after discharge (0/24 vs. 0/16, *p* = 1.000; Table 9). Further analysis was performed according to gestational age (Table 10), and the data showed that for preterm infants (*n* = 18; GA fluctuating between 30.14 and 36.86 weeks), the new tracheal tube not only shortened the duration of mechanical ventilation (101.75 ± 39.72 vs. 155.50 ± 51.49 h, *p* = 0.026; Table 10) and total ventilation (216.00 ± 81.60 vs. 351.50 ± 113.79 h, *p* = 0.010; Table 10), but also demonstrated significant advantages in reducing the duration of oxygen therapy (9.75 ± 6.02 vs. 17.33 ± 8.43 days, *p* = 0.042; Table 10); interestingly, however, there was no statistical difference in efficacy outcomes between the two groups in full-term infants (*n* = 22; GA fluctuating between 37.00 and 41.14 weeks) (*p* > 0.05; Table 10).

For mechanically ventilated infants without endotracheal surfactant administration (*n* = 23; GA fluctuating between 33.71 and 42.29 weeks), there were no significant differences between the two groups regarding any effectiveness indicators (*p* > 0.05; Table 9). Notably, all patients in this subgroup were full-term (*n* = 22; GA fluctuating between 37.29 and 42.29 weeks), except for one preterm infant with a GA of 33.71 weeks in the novel catheter group. Therefore, no further subgroup analysis was performed based on gestational age.

In the detailed analysis of the effectiveness of the new catheter in infants with the InSurE technique (*n* = 38; GA fluctuating between 28.00 and 37.57 weeks), no statistically significant differences were found (*p* > 0.05; Table 8). Notably, the duration of non-invasive ventilation appeared to be shorter in neonates who opted for the new tracheal tube (*n* = 20; GA fluctuating between 28.00 and 37.57 weeks) than in those with the conventional tube (*n* = 18; GA fluctuating between 28.00 and 37.14 weeks), although the difference was not statistically significant (92.50[62.00, 153.75] vs. 108.00[66.00, 200.25] hours, *p* = 0.884; Table 8). Similarly, subgroup analysis was performed based on the gestational age, and no statistical differences were found in either subgroup (*p* > 0.05; Table 11).

## Discussion

In general, based on the available data, it can be tentatively determined that such a design is effective and safe for neonates with RDS, both preterm and term, regardless of whether the newborn receives invasive mechanical ventilation or the InSurE technique and whether the tracheal tube serves as a bridge for ventilation or a route for drug delivery. Given the design philosophy of this dual-lumen catheter, the investigation of its safety and effectiveness focused on newborns requiring surfactant administration *via* the tracheal tube during mechanical ventilation. In this target population, the application of the new catheter significantly reduced the duration of mechanical ventilation, total ventilation, and oxygen therapy in preterm infants; however, such an advantage was not evident in term neonates. Moreover, no significant differences were found between the new and conventional tracheal tubes regarding safety outcomes, either in the full-term or preterm infants subgroup. Furthermore, in neonates mechanically ventilated without surfactant administration and in neonates receiving the InSurE technique, the dual-lumen tracheal tube showed similar safety and efficacy to conventional tracheal tubes. Given the design rationale for this catheter, such data are actually consistent with our expectations and suggest that the application of this new catheter may not be limited to neonates requiring endotracheal surfactant administration during mechanical ventilation, although it was designed primarily for this group.

Notably, the rate of intratracheal surfactant administration was significantly higher in the new tracheal tube group compared to the conventional tracheal tube group. Considering that the primary purpose of endotracheal intubation is different for neonates receiving mechanical ventilation and those with the InSurE technique, we performed a subgroup analysis accordingly. The data showed that for mechanically ventilated neonates, the proportion of preterm infants and the rate of surfactant administration were significantly greater in the new tracheal tube group than in the conventional tracheal tube group; while for neonates receiving the InSurE technique, no significant differences were found between the two groups. Similarly, a stratified analysis based on the purpose of intubation suggested that mechanically ventilated neonates were more likely to face endotracheal surfactant administration when a double-lumen tracheal tube was selected. Such data suggest that the double-lumen tracheal tube may lead to worse ventilation in mechanically ventilated neonates, which casts a shadow on the effectiveness and safety of the new catheter. However, it is unreasonable to attribute a higher surfactant administration rate to the use of new tracheal tubes without eliminating the effect of prematurity on the outcome. In this context, a stratified analysis according to gestational age (Premature and full-term) for mechanically ventilated neonates was born, and the data showed no significant association between the choice of the new tracheal tube and the need for endotracheal surfactant therapy after excluding the confounding factor of prematurity. Therefore, it is reasonable to suspect that the higher rate of endotracheal surfactant administration is related to the characteristics of the participants themselves. Nevertheless, given the statistical validity limited by the small sample size in this study, we still cannot completely exclude the risk of the new tracheal tube leading to worse ventilation or other poor outcomes. In further randomized controlled trials with large sample sizes, a targeted assessment of the various risks that may be associated with a dual-lumen tracheal tube is necessary.

Even with the popularity of minimally invasive and noninvasive surfactant administration technologies, mechanical ventilation combined with drug administration *via* endotracheal tube remains an irreplaceable treatment protocol. However, due to the limitations of the conventional tracheal tube, temporary interruptions of mechanical ventilation are unavoidable, which may lead to an increased risk of various adverse effects (28, 29). Our study confirmed that for neonates requiring endotracheal surfactant during mechanical ventilation, this dual-lumen tube could avoid interruption of ventilation and facilitate ventilator withdrawal while ensuring safety. Furthermore, the applicability of such a design was also demonstrated in other groups, including mechanically ventilated neonates without endotracheal surfactant therapy and neonates receiving the InSurE technique. If the clinical feasibility can be confirmed in further studies, such a design may have the potential to replace conventional catheters for volume use in various centers.

To our knowledge, this is the second prospective report on a double-lumen tracheal tube for newborns, while the first prospective study was provided by Valls-i-Soler et al. from Spain in 1998 (26). In the design of Valls-i-Soler et al., the drug delivery tube and the dominant tube are always separate and unconnected, with the end of the drug delivery tube opening directly into the trachea to minimize the possibility of drug wastage. However, such a design may present other drawbacks or risks, such as uneven drug distribution, direct irritation of the trachea due to the greater pressure generated by rapid drug injection, and the risk of blockage of the Murphy Eye. Notable, the Murphy Eye can provide an alternative route to maintain ventilation when the main opening of the catheter is blocked for various reasons. On balance, in our design, the opening of the drug delivery tube was designed to be at the upper end of the Murphy Eye, allowing the drug to enter the trachea in as precise a dose as possible while diluting the sputum around the Murphy Eye to relieve the blockage. Furthermore, with the buffering space provided by the Murphy eye, the direct irritation to the fragile airways of the newborn due to the greater pressure generated by rapid drug injection can be reduced. Moreover, the combined effect of the buffer space and the airflow in the main tube may allow for a relatively even distribution of the drug, preventing it from always entering the trachea along the same side of the inner wall. In addition, a plastic cap was designed to replace the valve as a closure for the drug injection port in order to reduce costs.

In the trial by Valls-i-Soler et al. (26), no statistical differences were found between the effectiveness of the two tracheal tubes, as measured by the setting of respiratory parameters, duration of mechanical ventilation, and the number of surfactant doses required. In contrast, the effectiveness demonstrated by our new tracheal tube is more impressive in a population with the same attributes, i.e., neonates requiring intratracheal surfactant administration during mechanical ventilation, especially premature neonates. The shortened duration of mechanical ventilation may be closely related to the design advantages of the dual-lumen catheter, although accurate and in-depth validation is not yet available. In addition, such a significant advantage may also be related to the success of the new catheter in avoiding temporary interruption of mechanical ventilation. In our study, neonates who opted for mechanical ventilation were usually in a more unstable state compared to those on non-invasive ventilation. When ventilation was suspended followed by surfactant administration in this situation, fluctuations in oxygen saturation cannot be completely avoided, even if the respiratory parameters were appropriately adjusted immediately after the operation. Therefore, although the duration of the endotracheal surfactant administration was limited to a few minutes, neonates with conventional catheters might still face an unstable internal environment and hemodynamics for a short period of time, which would increase the need for subsequent mechanical ventilation in this vulnerable population (30).

However, such an essential advantage of the new catheter failed to be demonstrated in full-term infants, which may be related to the fact that the drug administered intratracheally in our study was limited to surfactant. Unlike preterm neonates, the mechanism underlying RDS in term newborns is not dominated by a deficiency or dysfunction of pulmonary surfactant; therefore, term newborns are generally less responsive to surfactant therapy (31, 32). Nevertheless, it is unreasonable to interpret our results as an amplification of the efficacy of surfactant in preterm newborns by the new tracheal tube based on these speculations alone. Studies with larger sample sizes are needed to rule out the possibility that the effectiveness of the new tracheal tube itself varies by gestational age.

There is no precise conclusion on the optimal mode of intratracheal surfactant administration (33). However, a meta-analysis of preterm infants with respiratory distress syndrome showed that surfactant administration *via* a thin catheter reduced the risk of death and BPD compared with that *via* a tracheal tube (34). Besides, a net meta-analysis showed that among the various popular methods of endotracheal surfactant administration, surfactant administration *via* a fine catheter could show a more substantial advantage in reducing mortality, the need for mechanical ventilation, and the risk of BPD (35). Therefore, after validating the clinical applicability of the dual-lumen tracheal catheter, it is necessary to shift the focus of the study to a comparison between the dual-lumen tracheal catheter and the thin catheter to update the assessment of the optimal surfactant delivery method. However, it is necessary to emphasize differences in the target populations for tracheal tubes and thin catheters. While the thin catheter is primarily used in neonates with spontaneous breathing and stable hemodynamics (36), the advantages of the new dual-lumen catheter focus on neonates receiving mechanical ventilation, although still suitable for neonates requiring tracheal intubation in other situations. It is this difference that establishes the unassailable position of surfactant administration *via* the tracheal tube. Therefore, even though the surfactant administration *via* a thin catheter demonstrates a stronger advantage in comparison with the InSurE technique with a double-lumen tracheal tube, the effectiveness of the double-lumen tracheal tube for mechanically ventilated neonates remains irreplaceable.

Overall, there are still some limitations in our study. First of all, this is a single-center study from China, and its statistical validity was limited by the small sample size. Moreover, the study population was limited to neonates with RDS, and all drugs administered intratracheally were surfactants. Furthermore, no subgroup analysis was performed based on the severity of RDS. In our study, repeated administration of surfactant was determined flexibly by the clinician based on the condition of the newborn, so that neonates with more severe symptoms may receive more frequent surfactant treatment. From a theoretical point of view, the advantages of the dual-lumen catheter should have been maximized in this particular group, especially those on mechanical ventilation, but unfortunately, this idea was not reflected in our study. In addition, there was no long-term follow-up of respiratory and neurological function.

To address these limitations, further multicenter prospective randomized trials with large sample sizes and long-term follow-up of multisystem functions are being planned. Some detailed subgroup analysis can be performed on the basis of large sample sizes, and assessments for a specific population, such as those at high risk for repeat dosing (37, 38), can also be considered, which will help us to assess the target population and the applicable scope of this catheter. Besides, the expanded sample size should not be limited to neonates with RDS requiring surfactant therapy; other therapies administered *via* the tracheal tube or surfactant therapies for other diseases deserve to be further explored. What's more, neonates receiving both the InSurE technique and invasive mechanical ventilation will be included in further clinical trials to assess the impact of a dual-lumen tracheal tube on the success rate of the InSurE technique. In addition, the double-lumen tracheal tube can be compared with other new tracheal tubes, and the method of surfactant administration *via* the new tracheal tube can also be compared with other techniques of endotracheal surfactant administration, to allow for a tailored program for newborns when conditions permit.

## Conclusion

In general, preliminary evidence suggests that our new dual-lumen tracheal tube is safe and effective in neonates with RDS, either as an intermediary for ventilation or as a conduit for surfactant administration, but especially in preterm infants requiring intratracheal surfactant therapy during mechanical ventilation. Nevertheless, these results should be viewed with caution, given the limitations. Multicenter prospective randomized trials with large sample sizes are urgently needed for a more detailed assessment.

## Data Availability

The original contributions presented in the study are included in the article/**Supplementary Material**, further inquiries can be directed to the corresponding author/s.
