# Supplementary material for: Safety and efficacy of a novel double-lumen tracheal tube in neonates with RDS: A prospective cohort study
Source: Front Pediatr. 2022 Dec 5;10:1032044. doi: 10.3389/fped.2022.1032044 (PMC9760922; doi:10.3389/fped.2022.1032044)
Supplement: Supplementary file 1 [file Datasheet1.pdf]

frontiers

Supplementary Material

Supplementary Table 1. Information on the diameter and insertion depth of the double-lumen tracheal tube

**Model**	**Outer diameter of the dominant tube, mm**		**Inner diameter of the dominant tube, mm**		**Minimum depth of endotracheal intubation, mm**	
	**Nominal diameter**	**Actual diameter**	**Nominal diameter**	**Actual diameter**	**Via the nose**	**Via the mouth**
1	3.3	3.3±0.15	2.5	2.5±0.15	140	110
2	4.0	4.0±0.15	3.0	3.0±0.15	160	120
3	4.7	4.7±0.15	3.5	3.5±0.15	180	130
4	5.3	5.3±0.15	4.0	4.0±0.15	200	140

Supplementary Table2. Information on additional properties of the double-lumen tracheal tube

**Test Project**		**Test conditions**	**Environmental conditions in the laboratory**	**Result**	**Conclusion**
Shore hardness		Thickness of Specimen (3 layers stacked): 5.64 mm	Ambient temperature: 23 ± 2℃; Relative humidity: 50 ± 5%	A/1:90*	Qualified
Tensile test	Tensile strength	Width of the narrow part of the specimen: 5.99 mm; Thickness of Specimen: 1.178 mm; Testing speed: 250 mm/min; Gage length: 25 mm		23.2 MPa	Qualified
	Elongation at break			253%	Qualified
Thermal Stability Time(180℃)		Conditions: Oil bath, 180°C; Test strip: Congo Red.		260 min	Qualified

*A/1:90 means a hardness value of 90 for the sample, with a Type A hardness tester and a reading time of 1s.

Supplementary Table 3. Stratified analysis of basic information according to gestational age(Full-term, and preterm)

		Risk Estimate		Tests of Homogeneity of the Odds Ratio	Mantel-Haenszel Common Odds Ratio Estimate	
		**OR(95%CI)**	**p-value**	**p-value**	**OR(95%CI)**	**p-value**
Sex(male)	Full-term	1.032(0.319-3.341)	0.958	>0.05	0.822(0.372-1.814)	0.627
	Preterm	0.679(0.231-1.994)	0.480			
Cesarean	Full-term	0.529(0.161-1.744)	0.293	>0.05	0.435(0.182-1.040)	0.061
	Preterm	0.351(0.096-1.280)	0.105			
PROM	Full-term	1.500(0.268-8.383)	0.680	>0.05	0.916(0.352-2.383)	0.857
	Preterm	0.734(0.232-2.325)	0.598			
GDM	Full-term	NA	1.000	NA	NA	NA
	Preterm	NA	0.068			
Prenatal glucocorticoid	Full-term	NA	1.000	NA	0.509(0.165-1.572)	0.240
	Preterm	0.509(0.165-1.572)	0.238			
Placenta praevia	Full-term	NA	1.000	>0.05	0.508(0.075-3.421)	0.486
	Preterm	0.700(0.091-5.371)	1.000			
HDCP	Full-term	NA	1.000	>0.05	0.116(0.013-1.074)	0.058
	Preterm	0.116(0.013-1.074)	0.072			
ICP	Full-term	NA	1.000	NA	NA	NA
	Preterm	NA	1.000			
In vitro fertilization	Full-term	NA	1.000	NA	0.952(0.192-4.732)	0.952
	Preterm	0.952(0.192-4.732)	1.000			
Surfactant administration via endotracheal tube	Full-term	3.152(0.917-10.835)	0.064	>0.05	2.440(0.777-7.668)	0.127
	Preterm	NA	1.000			

* P <0.05; GDM, gestational diabetes mellitus; PROM, premature rupture of the membrane; HDCP; Hypertensive disorder complicating pregnancy; ICP, intrahepatic cholestasis of pregnancy.

Supplementary Table 4. Stratified analysis of basic information according to the purpose of intubation(Invasive mechanical ventilation, and the InSurE technique followed by noninvasive ventilation)

		**Risk Estimate**		**Tests of Homogeneity of the Odds Ratio**	**Mantel-Haenszel Common Odds Ratio Estimate**	
		**OR(95%CI)**	**p-value**	**p-value**	**OR(95%CI)**	**p-value**
Premature	Mechanical ventilation	3.130(1.002-9.774)	0.045*	>0.05	2.713(0.952-7.736)	0.062
	InSurE technique	1.118(0.065-19.283)	1.000			
Sex(male)	Mechanical ventilation	1.222(0.451-3.306)	0.693	>0.05	0.831(0.381-1.813)	0.642
	InSurE technique	0.431(0.117-1.592)	0.328			
Cesarean	Mechanical ventilation	0.559(0.203-1.540)	0.259	>0.05	0.492(0.212-1.145)	0.100
	InSurE technique	0.371(0.079-1.738)	0.278			
PROM	Mechanical ventilation	1.346(0.326-5.564)	0.732	>0.05	1.050(0.401-2.750)	0.921
	InSurE technique	0.846(0.226-3.167)	1.000			
GDM	Mechanical ventilation	NA	1.000	NA	NA	NA
	InSurE technique	NA	0.218			
Prenatal glucocorticoid	Mechanical ventilation	0.667(0.104-4.290)	1.000	>0.05	0.781(0.266-2.286)	0.651
	InSurE technique	0.846(0.226-3.167)	1.000			
Placenta praevia	Mechanical ventilation	NA	0.492	>0.05	0.614(0.096-3.933)	0.607
	InSurE technique	1.889(0.157-22.789)	1.000			
HDCP	Mechanical ventilation	NA	0.492	>0.05	0.159(0.017-1.514)	0.110
	InSurE technique	0.263(0.025-2.793)	0.328			
ICP	Mechanical ventilation	NA	1.000	NA	NA	NA
	InSurE technique	NA	1.000			
In vitro fertilization	Mechanical ventilation	NA	0.238	>0.05	1.270(0.267-6.040)	0.763
	InSurE technique	0.556(0.082-3.774)	0.653			
Surfactant administration via endotracheal tube	Mechanical ventilation	3.429(1.152-10.202)	0.024*	NA	3.429(1.152-10.202)	0.027*
	InSurE technique	NA	1.000			

* P <0.05; GDM, gestational diabetes mellitus; PROM, premature rupture of the membrane; HDCP; Hypertensive disorder complicating pregnancy; ICP, intrahepatic cholestasis of pregnancy.

Supplementary Table 5. Stratified analysis of basic information according to gestational age(Full-term, and preterm) for neonates receiving invasive mechanical ventilation

		Risk Estimate		Tests of Homogeneity of the Odds Ratio	Mantel-Haenszel Common Odds Ratio Estimate	
		**OR(95%CI)**	**p-value**	**p-value**	**OR(95%CI)**	**p-value**
Sex(male)	Full-term	1.250(0.374-4.177)	0.717	>0.05	1.111(0.396-3.120)	0.841
	Preterm	0.800(0.105-6.104)	1.000			
Cesarean	Full-term	0.500(0.148-1.694)	0.263	>0.05	0.452(0.153-1.336)	0.151
	Preterm	0.320(0.028-3.600)	0.605			
PROM	Full-term	1.533(0.273-8.627)	0.676	>0.05	1.308(0.307-5.577)	0.717
	Preterm	0.909(0.066-12.524)	1.000			
GDM	Full-term	NA	1.000	NA	NA	NA
	Preterm	NA	0.316			
Prenatal glucocorticoid	Full-term	NA	1.000	NA	0.182(0.020-1.638)	0.128
	Preterm	0.182(0.020-1.638)	0.262			
Placenta praevia	Full-term	NA	1.000	NA	NA	NA
	Preterm	NA	0.316			
HDCP	Full-term	NA	1.000	NA	NA	NA
	Preterm	NA	0.088			
ICP	Full-term	NA	1.000	NA	NA	NA
	Preterm	NA	1.000			
In vitro fertilization	Full-term	NA	1.000	NA	NA	NA
	Preterm	NA	1.000			
Surfactant administration via endotracheal tube	Full-term	3.200(0.909-11.268)	0.066	>0.05	2.598(0.798-8.457)	0.113
	Preterm	NA	1.000			

GDM, gestational diabetes mellitus; PROM, premature rupture of the membrane; HDCP; Hypertensive disorder complicating pregnancy; ICP, intrahepatic cholestasis of pregnancy.

Supplementary Table 6. Stratified analysis of basic information according to gestational age(Full-term, and preterm) for neonates receiving the InSurE technology

		Risk Estimate		Tests of Homogeneity of the Odds Ratio	Mantel-Haenszel Common Odds Ratio Estimate	
		**OR(95%CI)**	**p-value**	**p-value**	**OR(95%CI)**	**p-value**
Sex(male)	Full-term	NA	1.000	>0.05	0.444(0.124-1.592)	0.213
	Preterm	0.519(0.137-1.966)	0.503			
Cesarean	Full-term	NA	1.000	NA	0.367(0.077-1.743)	0.207
	Preterm	0.367(0.077-1.743)	0.274			
PROM	Full-term	NA	1.000	NA	0.833(0.218-3.190)	0.790
	Preterm	0.833(0.218-3.190)	1.000			
GDM	Full-term	NA	1.000	NA	NA	NA
	Preterm	NA	0.216			
Prenatal glucocorticoid	Full-term	NA	1.000	NA	0.833(0.218-3.190)	0.790
	Preterm	0.833(0.218-3.190)	1.000			
Placenta praevia	Full-term	NA	1.000	NA	1.882(0.155-22.832)	0.619
	Preterm	1.882(0.155-22.832)	1.000			
HDCP	Full-term	NA	1.000	NA	0.259(0.024-2.769)	0.264
	Preterm	0.259(0.024-2.769)	0.326			
ICP	Full-term	NA	1.000	NA	NA	NA
	Preterm	NA	1.000			
In vitro fertilization	Full-term	NA	1.000	NA	0.549(0.080-3.760)	0.541
	Preterm	0.549(0.080-3.760)	0.650			
Surfactant administration via endotracheal tube	Full-term	NA	1.000	NA	NA	NA
	Preterm	NA	1.000			

GDM, gestational diabetes mellitus; PROM, premature rupture of the membrane; HDCP; Hypertensive disorder complicating pregnancy; ICP, intrahepatic cholestasis of pregnancy.

Supplementary Table 7. Stratified analysis of adverse reactions according to gestational age(Full-term, and preterm)

		Risk Estimate		Tests of Homogeneity of the Odds Ratio	Mantel-Haenszel Common Odds Ratio Estimate	
		OR(95%CI)	p-value	p-value	OR(95%CI)	p-value
**Adverse reactions during intubation**						
Total	Full-term	NA	1.000	NA	NA	NA
	Preterm	NA	1.000			
Oral mucosal injury	Full-term	NA	1.000	NA	NA	NA
	Preterm	NA	1.000			
Bleeding airway injury	Full-term	NA	1.000	NA	NA	NA
	Preterm	NA	1.000			
Respiratory depression	Full-term	NA	1.000	NA	NA	NA
	Preterm	NA	1.000			
Bradycardia	Full-term	NA	1.000	NA	NA	NA
	Preterm	NA	1.000			
Tachycardia	Full-term	NA	1.000	NA	NA	NA
	Preterm	NA	1.000			
Arterial hypotension	Full-term	NA	1.000	NA	NA	NA
	Preterm	NA	1.000			
Laryngospasm	Full-term	NA	1.000	NA	NA	NA
	Preterm	NA	1.000			
Blockage of the drug delivery tube	Full-term	NA	1.000	NA	NA	NA
	Preterm	NA	1.000			
Distortion of the dominant tube	Full-term	NA	1.000	NA	NA	NA
	Preterm	NA	1.000			
**Adverse reactions after intubation**						
Total	Full-term	0.694(0.058-8.257)	1.000	>0.05	0.701(0.109-4.510)	0.708
	Preterm	0.710(0.042-11.968)	1.000			
Coughing and wheezing	Full-term	NA	1.000	NA	NA	NA
	Preterm	NA	1.000			
Recurrent choking on milk	Full-term	NA	1.000	NA	NA	NA
	Preterm	NA	1.000			
Pulmonary hemorrhage	Full-term	NA	1.000	NA	NA	NA

	Preterm	NA	1.000			
Emphysema	Full-term	NA	1.000	NA	NA	NA
	Preterm	NA	1.000			
IVH grade 3 or 4	Full-term	NA	1.000	NA	NA	NA
	Preterm	NA	0.418			
Secondary infection	Full-term	NA	0.504	>0.05	0.288(0.025-3.252)	0.314
	Preterm	0.710(0.042-11.968)	1.000			
Pneumothorax	Full-term	NA	0.413	NA	NA	NA
	Preterm	NA	1.000			
Concurrent laryngospasm	Full-term	NA	1.000	NA	NA	NA
	Preterm	NA	1.000			

IVH, Intraventricular hemorrhage.

Supplementary Table 8. Stratified analysis of adverse reactions according to the purpose of intubation(Invasive mechanical ventilation, and InSurE technique followed by noninvasive ventilation)

		Risk Estimate		Tests of Homogeneity of the Odds Ratio	Mantel-Haenszel Common Odds Ratio Estimate	
		OR(95%CI)	p-value	p-value	OR(95%CI)	p-value
**Adverse reactions during intubation**						
Total	Mechanical ventilation	NA	1.000	NA	NA	NA
	InSurE technique	NA	1.000			
Oral mucosal injury	Mechanical ventilation	NA	1.000	NA	NA	NA
	InSurE technique	NA	1.000			
Bleeding airway injury	Mechanical ventilation	NA	1.000	NA	NA	NA
	InSurE technique	NA	1.000			
Respiratory depression	Mechanical ventilation	NA	1.000	NA	NA	NA
	InSurE technique	NA	1.000			
Bradycardia	Mechanical ventilation	NA	1.000	NA	NA	NA
	InSurE technique	NA	1.000			
Tachycardia	Mechanical ventilation	NA	1.000	NA	NA	NA
	InSurE technique	NA	1.000			
Arterial hypotension	Mechanical ventilation	NA	1.000	NA	NA	NA
	InSurE technique	NA	1.000			
Laryngospasm	Mechanical ventilation	NA	1.000	NA	NA	NA
	InSurE technique	NA	1.000			
Blockage of the drug delivery tube	Mechanical ventilation	NA	1.000	NA	NA	NA
	InSurE technique	NA	1.000			
Distortion of the dominant tube	Mechanical ventilation	NA	1.000	NA	NA	NA
	InSurE technique	NA	1.000			
**Adverse reactions after intubation**						
Total	Mechanical ventilation	0.322(0.032-3.279)	0.613	>0.05	0.654(0.104-4.106)	0.650
	InSurE technique	NA	1.000			
Coughing and wheezing	Mechanical ventilation	NA	1.000	NA	NA	NA
	InSurE technique	NA	1.000			
Recurrent choking on milk	Mechanical ventilation	NA	1.000	NA	NA	NA
	InSurE technique	NA	1.000			

Pulmonary hemorrhage	Mechanical ventilation	NA	1.000	NA	NA	NA
	InSurE technique	NA	1.000			
Emphysema	Mechanical ventilation	NA	1.000	NA	NA	NA
	InSurE technique	NA	1.000			
IVH grade 3 or 4	Mechanical ventilation	NA	1.000	NA	NA	NA
	InSurE technique	NA	1.000			
Secondary infection	Mechanical ventilation	NA	0.238	>0.05	0.321(0.032-3.171)	0.331
	InSurE technique	NA	1.000			
Pneumothorax	Mechanical ventilation	NA	0.492	NA	NA	NA
	InSurE technique	NA	1.000			
Concurrent laryngospasm	Mechanical ventilation	NA	1.000	NA	NA	NA
	InSurE technique	NA	1.000			

IVH, Intraventricular hemorrhage.

Supplementary Table 9. Stratified analysis of adverse reactions according to gestational age(Full-term, and preterm) for neonates receiving invasive mechanical ventilation

		Risk Estimate		Tests of Homogeneity of the Odds Ratio	Mantel-Haenszel Common Odds Ratio Estimate	
		OR(95%CI)	p-value	p-value	OR(95%CI)	p-value
**Adverse reactions during intubation**						
Total	Full-term	NA	1.000	NA	NA	NA
	Preterm	NA	1.000			
Oral mucosal injury	Full-term	NA	1.000	NA	NA	NA
	Preterm	NA	1.000			
Bleeding airway injury	Full-term	NA	1.000	NA	NA	NA
	Preterm	NA	1.000			
Respiratory depression	Full-term	NA	1.000	NA	NA	NA
	Preterm	NA	1.000			
Bradycardia	Full-term	NA	1.000	NA	NA	NA
	Preterm	NA	1.000			
Tachycardia	Full-term	NA	1.000	NA	NA	NA
	Preterm	NA	1.000			
Arterial hypotension	Full-term	NA	1.000	NA	NA	NA
	Preterm	NA	1.000			
Laryngospasm	Full-term	NA	1.000	NA	NA	NA
	Preterm	NA	1.000			
Blockage of the drug delivery tube	Full-term	NA	1.000	NA	NA	NA
	Preterm	NA	1.000			
Distortion of the dominant tube	Full-term	NA	1.000	NA	NA	NA
	Preterm	NA	1.000			
**Adverse reactions after intubation**						
Total	Full-term	0.706(0.059-8.425)	1.000	>0.05	0.374(0.042-3.367)	0.381
	Preterm	NA	0.316			
Coughing and wheezing	Full-term	NA	1.000	NA	NA	NA
	Preterm	NA	1.000			
Recurrent choking on milk	Full-term	NA	1.000	NA	NA	NA
	Preterm	NA	1.000			

Pulmonary hemorrhage	Full-term	NA	1.000	NA	NA	NA
	Preterm	NA	1.000			
Emphysema	Full-term	NA	1.000	NA	NA	NA
	Preterm	NA	1.000			
IVH grade 3 or 4	Full-term	NA	1.000	NA	NA	NA
	Preterm	NA	0.316			
Secondary infection	Full-term	NA	0.505	NA	NA	NA
	Preterm	NA	0.316			
Pneumothorax	Full-term	NA	0.409	NA	NA	NA
	Preterm	NA	1.000			
Concurrent laryngospasm	Full-term	NA	1.000	NA	NA	NA
	Preterm	NA	1.000			

IVH, Intraventricular hemorrhage.

Supplementary Table 10. Stratified analysis of adverse reactions according to gestational age(Full-term, and preterm) for neonates receiving the InSurE technology

		Risk Estimate		Tests of Homogeneity of the Odds Ratio	Mantel-Haenszel Common Odds Ratio Estimate	
		OR(95%CI)	p-value	p-value	OR(95%CI)	p-value
**Adverse reactions during intubation**						
Total	Full-term	NA	1.000	NA	NA	NA
	Preterm	NA	1.000			
Oral mucosal injury	Full-term	NA	1.000	NA	NA	NA
	Preterm	NA	1.000			
Bleeding airway injury	Full-term	NA	1.000	NA	NA	NA
	Preterm	NA	1.000			
Respiratory depression	Full-term	NA	1.000	NA	NA	NA
	Preterm	NA	1.000			
Bradycardia	Full-term	NA	1.000	NA	NA	NA
	Preterm	NA	1.000			
Tachycardia	Full-term	NA	1.000	NA	NA	NA
	Preterm	NA	1.000			
Arterial hypotension	Full-term	NA	1.000	NA	NA	NA
	Preterm	NA	1.000			
Laryngospasm	Full-term	NA	1.000	NA	NA	NA
	Preterm	NA	1.000			
Blockage of the drug delivery tube	Full-term	NA	1.000	NA	NA	NA
	Preterm	NA	1.000			
Distortion of the dominant tube	Full-term	NA	1.000	NA	NA	NA
	Preterm	NA	1.000			
**Adverse reactions after intubation**						
Total	Full-term	NA	1.000	NA	NA	NA
	Preterm	NA	1.000			
Coughing and wheezing	Full-term	NA	1.000	NA	NA	NA
	Preterm	NA	1.000			
Recurrent choking on milk	Full-term	NA	1.000	NA	NA	NA
	Preterm	NA	1.000			

Pulmonary hemorrhage	Full-term	NA	1.000	NA	NA	NA
	Preterm	NA	1.000			
Emphysema	Full-term	NA	1.000	NA	NA	NA
	Preterm	NA	1.000			
IVH grade 3 or 4	Full-term	NA	1.000	NA	NA	NA
	Preterm	NA	1.000			
Secondary infection	Full-term	NA	1.000	NA	NA	NA
	Preterm	NA	1.000			
Pneumothorax	Full-term	NA	1.000	NA	NA	NA
	Preterm	NA	1.000			
Concurrent laryngospasm	Full-term	NA	1.000	NA	NA	NA
	Preterm	NA	1.000			

IVH, Intraventricular hemorrhage.

Supplementary Table 11. Stratified analysis of the efficacy of the tracheal tube according to gestational age(Full-term, and preterm) or the purpose of intubation(Invasive mechanical ventilation, and InSurE technique followed by noninvasive ventilation)

		**Risk Estimate**		**Tests of Homogeneity of the Odds Ratio**	**Mantel-Haenszel Common Odds Ratio Estimate**	
		**OR(95%CI)**	**p-value**	**p-value**	**OR(95%CI)**	**p-value**
**According to gestational age(Full-term, and preterm)**						
Discharged with oxygen	Full-term	1.533(0.332-7.088)	0.700	>0.05	1.084(0.451-2.606)	0.856
	Preterm	0.917(0.314-2.678)	0.874			
Recurrent dyspnea one month after discharge	Full-term	NA	1.000	NA	NA	NA
	Preterm	NA	1.000			
**According to the purpose of intubation(Invasive mechanical ventilation, and InSurE technique)**						
Discharged with oxygen	Mechanical ventilation	1.242(0.389-3.970)	0.714	>0.05	1.352(0.571-3.200)	0.493
	InSurE technique	1.500(0.415-5.428)	0.745			
Recurrent dyspnea one month after discharge	Mechanical ventilation	NA	1.000	NA	NA	NA
	InSurE technique	NA	1.000			

Supplementary Table 12. Stratified analysis of the efficacy of the tracheal tube according to gestational age(Full-term, and preterm) for neonates receiving invasive mechanical ventilation and those with the InSurE technology

		Risk Estimate		Tests of Homogeneity of the Odds Ratio	Mantel-Haenszel Common Odds Ratio Estimate	
		OR(95%CI)	p-value	p-value	OR(95%CI)	p-value
**Newborns receiving invasive mechanical ventilation**						
Discharged with oxygen	Full-term	1.571(0.337-7.326)	0.697	>0.05	0.977(0.294-3.248)	0.970
	Preterm	0.444(0.061-3.242)	0.617			
Recurrent dyspnea one month after discharge	Full-term	NA	1.000	NA	NA	NA
	Preterm	NA	1.000			
**Newborns receiving the InSurE technique**						
Discharged with oxygen	Full-term	NA	1.000	NA	1.524(0.402-5.777)	0.536
	Preterm	1.524(0.402-5.777)	0.736			
Recurrent dyspnea one month after discharge	Full-term	NA	1.000	NA	NA	NA
	Preterm	NA	1.000			
